# Effect of Nanoemulsions of *Chrysanthemum cinerariaefolium* and *Oenothera biennis* Flowers Against the West Nile Virus Vector, *Culex pipiens*, with Phytochemical Profile

**DOI:** 10.3390/insects17090925

**Published:** 2026-09-03

**Authors:** Mohamed M. Baz, Lamya Ahmed Alkeridis, Laila Ali M. Al-shuraym, Hend M. Alharbi, Abdelfattah Selim, Esraa A. Elhawary

**Affiliations:** 1 Department of Entomology, Faculty of Science, Benha University, Benha 13518, Egypt; 2 Department of Biology, Faculty of Education and Arts, Sohar University, Sohar 311, Oman; 3 Department of Biology, Collage of Science, Princess Nourah bint Abdulrahman University, Riyadh 11671, Saudi Arabia; 4 Department of Animal Medicine (Infectious Diseases), College of Veterinary Medicine, Benha University, Toukh 13736, Egypt; 5 Department of Pharmacognosy, Faculty of Pharmacy, Ain-Shams University, Cairo 11566, Egypt; esraa.elhawary@pharma.asu.edu.eg

**Keywords:** *Chrysanthemum cinerariaefolium*, *Culex pipiens*, larvicidal activity, nanoemulsions, *Oenothera biennis*, phytochemical profile

## Abstract

(1) Background: Mosquitoes represent major vectors of human pathogens, and their control remains a global public health priority. However, the widespread use of synthetic insecticides has resulted in environmental contamination, adverse effects on non-target organisms, and the rapid emergence of insecticide resistance. These limitations have intensified the search for sustainable botanical alternatives. (2) Methods: The present study investigated the phytochemical composition and larvicidal efficacy of flower extracts from *Chrysanthemum cinerariaefolium* and *Oenothera biennis*, alongside their nanoemulsion formulations, against the third-instar larvae of *Culex pipiens*. Larvicidal assays demonstrated that nanoemulsion formulations markedly enhanced bioactivity compared with conventional extracts. (3) Results: After 48 h of exposure, *C. cinerariaefolium* nanoemulsion exhibited the strongest toxicity (LC_50_ = 26.63 ppm), followed by methanol (44.46 ppm), hexane (73.90 ppm), and aqueous extracts (209.99 ppm). Corresponding LC_50_ values for *O. biennis* were 31.89, 80.15, 140.69, and 393.22 ppm, respectively. Biochemical analyses indicated that *C. cinerariaefolium* treatments strongly inhibited detoxification and digestive enzymes (esterases, GABA-T, amylase, and lipase) and suppressed antioxidant defenses (SOD, GST, CAT, and GSH). This was accompanied by a significant elevation of oxidative stress biomarkers, including lipid peroxidation and protein carbonylation. In contrast, *O. biennis* induced comparatively moderate biochemical responses. The phytochemical profiling utilizing GC-MS revealed the identification of forty-nine and fifty volatile components from the *C. cinerariaefolium* (CW-Hex) and *O. biennis* (BOF) n-hexane extracts, respectively, primarily consisting of oxygenated hydrocarbons, fatty acids, and their esters. Furthermore, UPLC-MS/MS-assisted profiling of methanol, ethyl acetate, and aqueous extracts identified thirty-eight metabolites for CW and thirty-one for BOF. The CW extracts were predominant in flavonoids and fatty acids while also containing cinnamic acid derivatives, phenolic acids, diterpenoids, triterpenoids, pyrethrins, phenyl ethanoids, steroids, aurones, and tannins. BOF extracts were particularly rich in flavonoids, followed by fatty acids, cinnamic acid derivatives, tannins, and steroids. (4) Conclusions: These findings demonstrate that plant-derived formulations of *C. cinerariaefolium*, particularly in nanoemulsion form, exert potent larvicidal activity through the disruption of metabolic and antioxidant pathways, offering a promising eco-friendly strategy for vector control.

## Simple Summary

This study evaluated flower extracts of *Chrysanthemum cinerariaefolium* and *Oenothera biennis* against *Culex pipiens* larvae. Nanoemulsion formulations showed stronger larvicidal activity, particularly the *C. cinerariaefolium* nanoemulsion (LC_50_ = 26.63 ppm). The treatments disrupted key metabolic and antioxidant enzymes in mosquito larvae. These findings highlight *C. cinerariaefolium* nanoemulsion as a promising eco-friendly alternative for mosquito control.

## 1. Introduction

Mosquitoes of the *Culex pipiens* complex are globally recognized as important vectors of several arboviral and parasitic diseases that affect both humans and animals. They are responsible for the transmission of West Nile virus (WNV), Rift Valley fever virus (RVFV), St. Louis encephalitis virus (SLEV), and Usutu virus (USUV), as well as filarial nematodes such as *Wuchereria bancrofti*, the causative agent of lymphatic filariasis [1,2,3]. These pathogens pose substantial health and economic challenges, particularly across Africa, the Middle East, and southern Europe, where *Cx. pipiens* populations thrive under favorable climatic conditions that promote transmission [4]. The public health burden is further exacerbated by the rapid urbanization and ecological adaptations of these vectors, necessitating more resilient management strategies that incorporate integrated pest management, community engagement, and sustainable practices to effectively address the challenges posed by these diseases.

To stop the spread of arboviral and filarial diseases, it is still important to keep *Cx. pipiens* populations under control. However, although synthetic insecticides have been highly effective in the short term, their extensive use has led to resistance development, environmental contamination, and harmful effects on non-target organisms [4,5,6]. This chemical dependency has created an ecological imbalance, driving the scientific community to seek “green” alternatives that align with integrated pest management (IPM) principles.

The growing limitations of conventional chemical insecticides, including resistance development, environmental hazards, and toxicity to non-target species, have prompted increasing interest in sustainable, plant-based alternatives [7]. Botanical insecticides contain diverse bioactive constituents such as alkaloids, terpenoids, flavonoids, and phenolics, which exhibit strong larvicidal, pupicidal, and adulticidal activities against mosquitoes [8,9]. These natural agents are biodegradable, environmentally safe, and act through multiple biochemical pathways, thereby minimizing the risk of resistance development [10].

Among various botanical sources, *Chrysanthemum cinerariaefolium* and *Oenothera biennis* have attracted increasing attention for their insecticidal potential. *C. cinerariaefolium* (pyrethrum or *Dalmatian chrysanthemum*), a member of the family Asteraceae, is a natural source of pyrethrin esters of chrysanthemic and pyrethric acids that act on insect sodium channels, causing rapid knockdown and death [11]. These compounds are among the most effective and environmentally friendly bioinsecticides [12]. Complementing this, *O. biennis* (evening primrose), belonging to the family Onagraceae, contains a wide range of phenolics, flavonoids, and polyunsaturated fatty acids such as γ-linolenic acid, which display antioxidant, antimicrobial, and insecticidal properties [13]. Utilizing such chemically distinct plant extracts may provide synergistic toxicity by targeting different physiological systems in the larvae while maintaining ecological safety.

The efficiency of phytochemical and antioxidant extraction from plant materials depends on several factors, including extraction time, temperature, and solvent properties such as concentration and polarity. Since phytochemicals differ in polarity and solubility, no single solvent can effectively extract all bioactive constituents. Therefore, a serial exhaustive extraction approach is often applied, using solvents of increasing polarity from non-polar (e.g., *n*-hexane) to polar (e.g., water) to achieve comprehensive recovery of both lipophilic and hydrophilic metabolites [14]. Previous studies have shown that solvent polarity significantly affects not only extraction yield but also the antioxidant and biological activities of plant phenolics [15]. This methodical fractionation is crucial for pinpointing the specific chemical classes responsible for larval mortality.

Nevertheless, limitations related to solubility, volatility, and stability often reduce the practical effectiveness of conventional botanical extracts in aquatic habitats. Nanotechnology has recently emerged as a powerful tool in vector control to overcome these challenges. It involves manipulating materials at the nanoscale, typically between 0.1 and 200 nanometers, to enhance the effectiveness of pest control methods. Nanoemulsion-based systems enhance the solubility, stability, and bioavailability of hydrophobic phytochemicals, enabling controlled release and prolonged residual activity. Nanomaterials (0.1–200 nm) possess unique physicochemical properties that facilitate targeted delivery and controlled release, thereby improving efficacy and minimizing environmental impact. Consequently, nanoemulsions of plant extracts offer a promising, eco-friendly, and cost-effective strategy for managing vector-borne diseases [16].

Given the urgent need for sustainable mosquito control strategies, investigating the insecticidal potential of *Chrysanthemum cinerariaefolium* and *Oenothera biennis* within nanoemulsion formulations represents a promising approach. The action of pyrethrins from *C. cinerariaefolium* and phenolic constituents from *O. biennis* may enhance larvicidal efficacy while reducing environmental toxicity.

Earlier, we reported the potential of using nanoemulsions of *Araucaria heterophylla* and *Azadirachta indica* for controlling *Culex pipiens* mosquitoes, and these were very effective against mosquito larvae [17,18,19]. However, the present study broadens the scope of research to assess the larvicidal efficacy of two different plants, namely *C. cinerariaefolium* and *O. biennis*, due to their dissimilar phytochemical constitution. Furthermore, the present study provides a detailed comparative phytochemical analysis by employing GC-MS and UPLC-MS/MS, allowing the association of specific bioactive compounds, such as pyrethrins present in *C. cinerariaefolium,* with biochemical disturbances and oxidative stress in larvae. This offers a better understanding of their mechanisms of action in disrupting insect physiology.

Therefore, the present study aims to evaluate the larvicidal efficacy of several *C. cinerariaefolium* and *O. biennis* extracts and their corresponding nanoemulsions against *Cx. pipiens*, a major vector of the West Nile virus. Additionally, the study seeks to characterize the phytochemical profiles of these plants using advanced analytical techniques (e.g., GC-MS or HPLC) to identify the bioactive constituents responsible for their insecticidal activity. This integrative approach is expected to provide new insights into eco-friendly vector management and support the development of sustainable botanical nano-biopesticides.

## 2. Materials and Methods

### 2.1. Chemicals

Butyl alcohol, stearic acid, lauric acid, oleic acid, Tween 20, sodium glycocholate, and 50% hydrolyzed ammonium hydroxide were purchased from Alfa Aesar Germany (Thermo Fisher Scientific, Kandel, Germany). Methyl alcohol was obtained from El-Gomhoria Company (Cairo, Egypt) and distilled water were prepared in our lab using two stages of high-purity distillation (GFL 2004, Burgwedel, Germany). All solvents and chemicals were used without any further purification.

### 2.2. Plant Materials and Analysis

#### 2.2.1. Plant Collection

Flowers of *Chrysanthemum cinerariaefolium* and *Oenothera biennis* were procured from Harraz for Medicinal Herbs, Food Industries, and Natural Products Company (Cairo, Egypt). The botanical identity of the specimens was confirmed by Dr. Trease Labib, a plant taxonomist affiliated with the Egyptian Ministry of Agriculture.

#### 2.2.2. Plant Extraction

The collected flowers were shade-dried at room temperature (27 ± 2 °C) and finely powdered using a stainless-steel electric grinder. The obtained powder was stored in airtight containers to prevent moisture uptake. Extractions were performed using a Soxhlet apparatus using three solvents: methanol (65 °C), hexane (69 °C), and water (100 °C). For each solvent, 40 g of the powdered material was extracted with 200 mL of solvent for 5 to 12 h depending on the solvent’s boiling point and exhaustion of the plant material. The resulting extracts were concentrated under reduced pressure using a rotary evaporator at a temperature not exceeding 40 °C to preserve thermolabile constituents and stored in dark glass bottles to avoid light-induced degradation [20].

### 2.3. Preparation of Nanoemulsions

Nanoemulsion formulations were prepared following the homogenization protocol described by Radwan et al. [21] with minor modifications. The procedure consisted of mixing equal volumes (2.5 mL) of plant extract and oleic acid in a glass beaker (named B1). The mixture was heated at 45 °C. In a separate beaker (B2), the aqueous phase was prepared by adding 10 mL of distilled water, 0.2 g of sodium glycocholate, 0.25 mL of butanol, and 3 mL of polysorbate 20 and stirring continuously on a hot plate until complete dissolution. The temperature of both phases was maintained at 45 °C and monitored by an infrared thermometer. The oil phase (B1) was then added slowly to the aqueous phase (B2) under constant stirring at the same temperature to form the primary coarse emulsion. Finally, the nanoemulsion was rapidly cooled by immersion in an ice bath and diluted to a final volume of 40 mL with distilled water. The obtained nanoemulsion was transferred to a 50 mL Falcon tube and stored under refrigerated conditions until further use.

### 2.4. Characterization of Plant Extract Nanoemulsion

#### 2.4.1. Average Droplet Size and Surface Charge

The mean droplet diameter, average particle size, and polydispersity index (PDI) of the prepared nanoemulsions were determined using the dynamic light scattering (DLS) technique. Measurements were performed at room temperature with a fixed scattering angle of 173°. The zeta potential (ζ-potential), representing the net surface charge of the droplets, was recorded by monitoring the frequency shift of the scattered light at a 12° angle during laser beam irradiation. All measurements of droplet size, PDI, and zeta potential were conducted using a Zetasizer Nano ZS instrument (Malvern Instruments Ltd., Malvern, UK) at the Egyptian Petroleum Research Institute (EPRI). To avoid multiple scattering effects, approximately 5–10 mg of each sample was dispersed in 10 mL of distilled water at 25 °C and homogenized for two minutes prior to measurement [22]. Each measurement was performed in triplicate to ensure reproducibility.

#### 2.4.2. Nanoemulsion Droplet Morphology by Transmission Electron Microscopy (TEM)

The morphology and internal architecture of the nanoemulsion droplets were examined using high-resolution transmission electron microscopy (HR-TEM; JEOL JEM-2100-115, Tokyo, Japan) at the central laboratories of the Egyptian Petroleum Research Institute (EPRI), Cairo. Imaging was performed at an accelerating voltage of 200 kV. For sample preparation, 1 µL of the nanoemulsion was diluted with distilled water (1:200) and placed on a 200-mesh carbon-coated copper grid for 2 min. Excess liquid was removed with cellulose filter paper. Subsequently, one to two drops of 2% (*w*/*w*) phosphotungstic acid (PTA) were added for 10 s to achieve negative staining, and any residual stain was carefully absorbed using filter paper before imaging [23]. This technique provided a clear visualization of the spherical shape and size distribution of the droplets, corroborating the DLS data.

#### 2.4.3. UV-Vis Spectroscopic Analysis

To verify the proper formation of the nanoemulsions and examine their optical characteristics, we employed a Genway spectrophotometer 6305 (Genway Instruments, Tokyo, Japan) for UV analysis. At room temperature, the absorption spectra were scanned in the 200–800 nm region against a distilled water blank to confirm the successful incorporation of phytochemicals within the nano-droplets [24].

### 2.5. Mosquito Larvicidal Assay

#### 2.5.1. Rearing of *Culex pipiens*

Larvae of *Culex pipiens* were maintained under controlled insectary conditions at 27 ± 2 °C, with a relative humidity of 75 ± 5% and a 12:12 h light–dark photoperiod. The larvae were fed a diet composed of TetraMin fish food and finely ground bread in a ratio of 3:1. Upon pupation, individuals were transferred from enamel trays into containers filled with dechlorinated water and placed inside mesh cages (35 × 35 × 40 cm^3^) to allow adult emergence. Female adults were provided with periodic blood meals from a hamster, while both males and females were supplied continuously with a 10% sucrose solution. All bioassays were conducted within the same insectary, utilizing larvae derived from this colony [25].

#### 2.5.2. Larvicidal Activity

The larvicidal efficacy of methanolic, hexane, and aqueous flower extracts of *C. cinerariaefolium* and *O. biennis*, along with their nanoemulsion formulations, was evaluated against third-instar larvae of *Cx. pipiens* under laboratory conditions. Serial concentrations of each extract (25, 50, 125, 250, 500, and 1000 ppm; equivalent to 1 g per 1000 mL of distilled water) were tested. For each concentration, three replicates of 25 third-instar larvae were placed in glass beakers containing 250 mL of dechlorinated water with the corresponding extract. Mortality was assessed after 24 and 48 h of exposure, while control groups received 1 mL of ethanol only. The mortality rate in control treatments never exceeded 4% [26].

### 2.6. Biochemical and Oxidative Stress Analyses

The physiological and biochemical changes induced by the tested treatments were investigated using a full panel of enzymatic and non-enzymatic biomarkers in *Cx. pipiens* larvae after 24 h of exposure to the LC_50_ concentrations of the methanolic extracts and nanoemulsions of *C. cinerariaefolium* and *O. biennis*. The parameters assessed were major detoxification enzymes, namely acetylcholinesterase (AChE), α-esterase (α-EST), *β*-esterase (*β*-EST), and glutathione S-transferase (GST); and the non-enzymatic antioxidant reduced glutathione (GSH). To assess the extent of oxidative damage, oxidative stress markers, superoxide dismutase (SOD), catalase (CAT), lipid peroxidation (LPO), and total protein carbonyl content (TPC) were quantified. The activity of gamma-aminobutyric acid transaminase (GABA-T) and digestive enzymes (amylase and lipase) was also analyzed to demonstrate potential alterations of metabolic and physiological functions as described in previous protocols [27].

#### 2.6.1. Preparation of Tissue Homogenates

Third-instar larvae of *Cx. pipiens* were used for biochemical assays. Untreated larvae served as controls, while treated groups were exposed to the LC_50_ concentrations of each methanolic and nanoemulsion extract for 24 h. The larvae were homogenized in liquid nitrogen and suspended in chilled 50 mM sodium phosphate buffer (pH 7). Homogenization was carried out at approximately 4 °C using a manual homogenizer. The resulting homogenates were centrifuged at 10,000× *g* for 10 min at 4 °C, and the supernatant was collected for enzymatic activity determination.

#### 2.6.2. Acetylcholinesterase (AChE) Activity

AChE activity was quantified using a modified Ellman’s method [28] with 0.3 mM DTNB and 0.3 mM acetylthiocholine iodide as substrates. The absorbance was monitored at 412 nm for 5 min at 30 °C. Enzyme activity was expressed as µmol of substrate hydrolyzed per minute per mg of protein.

#### 2.6.3. α- and β-Esterase (α/β-EST) Activity

Total esterase activity was determined according to Penilla et al. [29] using 96-well microplates. α-naphthyl acetate and β-naphthyl acetate were used as substrates for α-EST and *β*-EST, respectively. The reaction was stopped by adding a coupling reagent (fast blue RR salt). Enzyme activity was expressed as µmol of product formed per minute per mg of protein. Protein concentration was determined following the Bradford method [30] using bovine serum albumin (BSA) as a standard.

#### 2.6.4. Gamma-Aminobutyric Acid Transaminase (GABA-T) Activity

GABA-T activity was measured based on the procedure of Boer and Bruinvels [31]. The reaction mixture contained Tris-HCl buffer, α-ketoglutarate, *β*-NAD, and 2-mercaptoethanol. The reaction was initiated by the addition of GABA, and the absorbance was recorded at 340 nm after 30 min of incubation at 25 °C.

#### 2.6.5. Digestive Enzymes (Amylase and Lipase) Activity

Amylase activity was determined following the method of Ishaaya and Swirski [32] using soluble starch as a substrate. The reaction was terminated by DNSA reagent, and absorbance was measured at 540 nm. Lipase activity was assayed following Itaya [33] using a substrate composed of gum acacia and olive oil. The absorbance was measured at 550 nm following the addition of chloroform and color reagent.

#### 2.6.6. Oxidative Stress Biomarkers

Superoxide dismutase (SOD) activity was determined according to Nishikimi et al. [34] by monitoring the inhibition of nitroblue tetrazolium (NBT) reduction. Catalase (CAT) activity was measured according to Aebi [35] by observing the decrease in absorbance of hydrogen peroxide (H_2_O_2_) at 240 nm. Glutathione S-transferase (GST) activity was analyzed according to Habig et al. [36] using CDNB and reduced glutathione (GSH) as substrates. Lipid peroxidation (LPO) was quantified as malondialdehyde (MDA) equivalents according to Ohkawa et al. [37]. Finally, total protein carbonyl (TPC) content was quantified as described by Levine [38] to assess oxidative protein damage.

### 2.7. Phytochemical Analysis

#### 2.7.1. GCMS Analysis

The *n*-hexane extract of the *Chrysanthemum cinerariaefolium* and *Oenothera biennis* flowers was injected into a gas chromatography–mass spectrometry instrument (Shimadzu GCMS-QP 2010, Kyoto, Japan) operating in electron impact (EI) mode at 70 eV, with mass spectra recorded across a range of 35–500 amu. Separation was performed using an Rxi-1MS capillary column (30 m × 0.25 mm i.d. × 0.25 µm film thickness; Restek, Bellefonte, PA, USA). A 1 µL volume was introduced using split mode at a split ratio of 30:1. The oven temperature profile began with an initial hold at 50 °C for 3 min, ramped to 300 °C at 5 °C/min, and concluded with an isothermal hold at 300 °C for 10 min. Helium was utilized as the carrier gas with a flow rate of 1.37 mL/min. Both the injector port and GC-MS interface temperatures were set to 280 °C, and the ion source was maintained at 200 °C. Retention indices (RI) were determined relative to a homologous series of n-alkanes (C8–C30) run under identical chromatographic conditions. Constituent identification was accomplished by matching the acquired mass spectra and calculated retention indices with data from the NIST-17 and Wiley reference libraries [39].

#### 2.7.2. UPLC/MS Analysis

The UPLC/ESI/MS analysis was performed for *Chrysanthemum cinerariaefolium* and *Oenothera biennis* methanol, ethyl acetate, and aqueous flower extracts using the method of [5,6,7,8]. UPLC/ESI/MS in both positive and negative ion acquisition modes was carried out on a XEVO TQD triple quadrupole mass spectrometer instrument (Waters Corporation, Milford, MA, USA). Chromatographic separation of the sample was done by injecting 10 μL into a UPLC instrument equipped with a reverse-phase C-18 column (ACQUITY UPLC—BEH, 2.1 × 50 mm column; 1.7 μm particle size). The sample solution (100 μg/mL) was prepared using HPLC-grade methanol, filtered using a membrane disc filter (0.2 μm), and degassed by sonication before injection, and then subjected to LC/ESI/MS analysis. The gradient mobile phase comprised two eluents: eluent A was H_2_O acidified with 0.1% formic acid, and eluent B was MeOH acidified with 0.1% formic acid. Elution was made at a flow rate of 0.2 mL/min as follows: (10% B) from 0 to 5 min; (30% B) from 5 to 15 min; (70% B) from 15 to 22 min; (90% B) from 22 to 25 min, and (100% B) from 25 to 29 min. The analysis was accomplished using negative ion mode as follows: source temperature 150 °C, cone voltage 30 eV, capillary voltage 3 kV, desolvation temperature 440 °C, cone gas flow 50 L/h, and desolvation gas flow 900 L/h. Mass spectra were recorded in Electrospray ionization (ESI) (negative and positive ion modes) (*m*/*z* 100–1000). UPLC/MS data were processed using MassLynx 4.1 software, and tentative identification was done by comparing their retention times (Rt), mass spectra with reported data.

#### 2.7.3. Multivariate Data Analysis

Unsupervised principal component analysis (PCA) was performed using Unscrambler X 10.3 (CAMO SA, Oslo, Norway). A clustered heat map was built using NCSS12 software with Euclidean distance and the unweighted pair group method [10,11].

### 2.8. Statistical Analysis

Data were expressed as mean ± standard deviation (SD) and analyzed via one-way analysis of variance (ANOVA) followed by Tukey’s multiple range test (*p* < 0.05) using SPSS v.22.0. The normality of distribution and homogeneity of variance were verified using Shapiro–Wilk and Kolmogorov–Smirnov tests. Probit analysis was employed to estimate the lethal concentrations (LC_50_, LC_90_, and LC_95_) along with their 95% confidence limits. All bioassays and biochemical experiments were conducted in at least three independent replicates. Furthermore, unsupervised principal component analysis (PCA) was performed using Unscrambler X 10.3 (CAMO SA, Oslo, Norway), and a clustered heat map was generated using NCSS 12 software based on Euclidean distance and the unweighted pair group method (UPGMA) to visualize phytochemical variations among the tested extracts and nanoemulsions.

## 3. Results

### 3.1. Characterization of Chrysanthemum cinerariaefolium and Oenothera biennis Nanoemulsions

#### 3.1.1. UV Spectroscopy Analysis of Nanoemulsions

**Figure 1:**
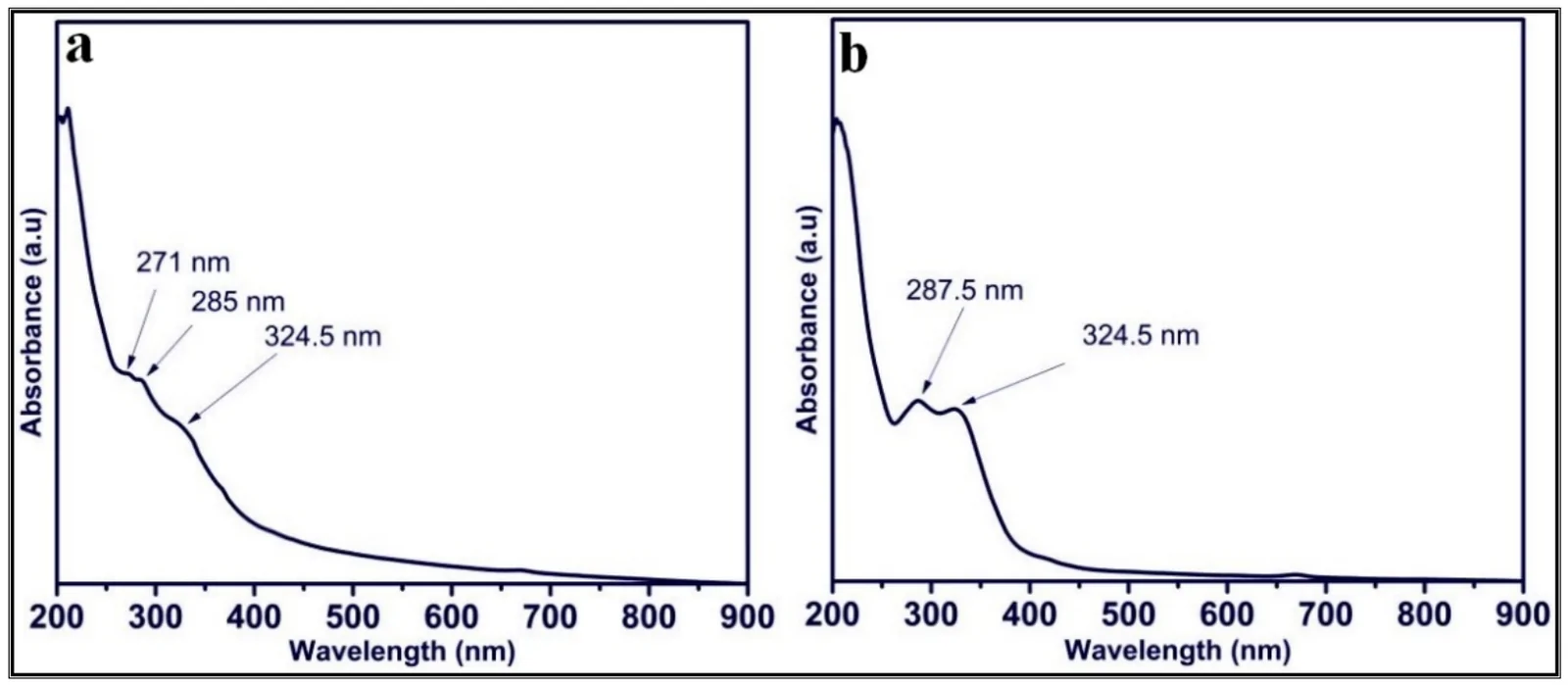
Ultraviolet-visible (UV-VIS) spectroscopy of *Chrysanthemum cinerariaefolium* (**a**) and *Oenothera biennis* (**b**) nanoemulsion.

The formation of nanoemulsions was confirmed by UV–Vis absorption spectra of nanoemulsions and encapsulated bioactive phytoconstituents (Figure 1). The absorption spectrum of *C. cinerariaefolium* nanoemulsion (Figure 1a) presented two major peaks at 271 nm and 285 nm with a shoulder peak at 324.5 nm. The strong band at 285 nm is of particular interest, showing successful encapsulation of the active ingredients of the extract in the nano-droplet system. This confirms that the bioactive substances are really dispersed at the nanoscale and not simply suspended as insoluble aggregates [40]. The spectrum of *O. biennis* nanoemulsion (Figure 1b) showed the typical absorption maximum at 287.5 nm and a shoulder at about 324.5 nm. The peak at 287.5 nm is the fingerprint of a successful nanoformulation due to specific phytochemical constituents of *O. biennis* extract that were successfully incorporated into the nanoemulsion droplets [41]. The spectral features are characteristic of stable nanoemulsions with good dispersion of bioactive components, which confirms the effectiveness of the nanoencapsulation process on both plant extracts.

#### 3.1.2. DLS Analysis and Size Distribution

**Figure 2:**
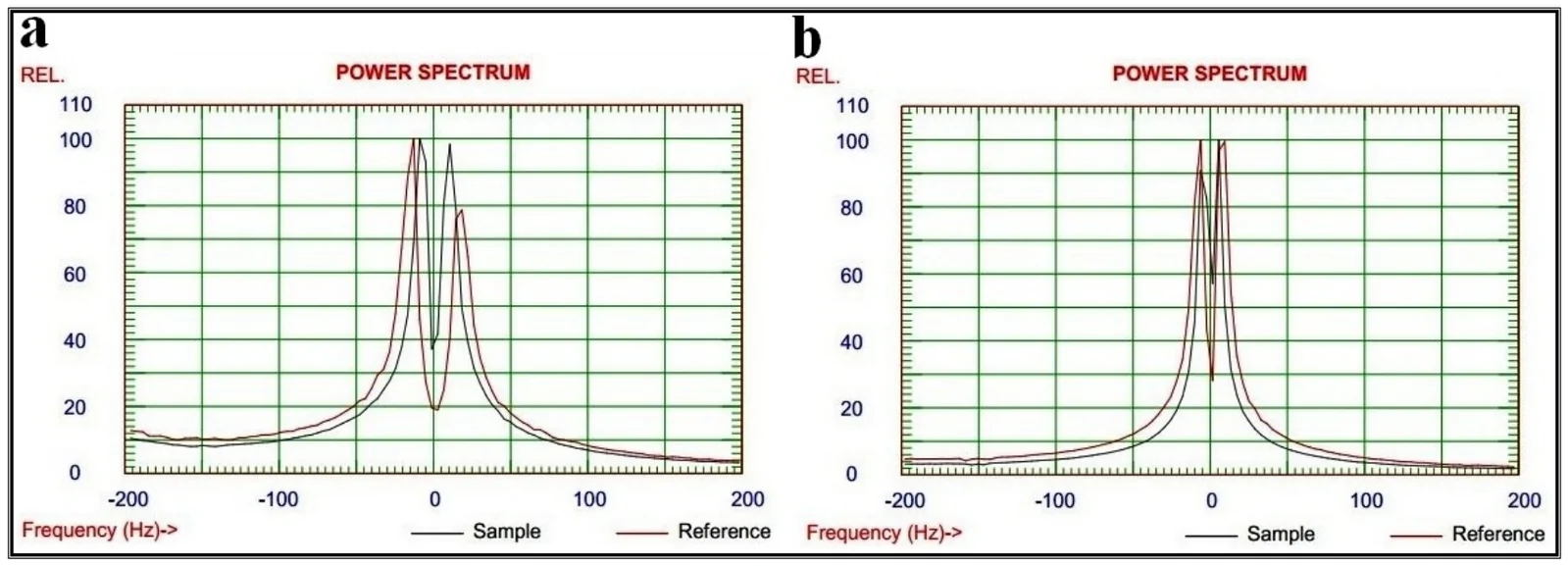
Dynamic light scattering of *Chrysanthemum cinerariaefolium* (**a**) and *Oenothera biennis* (**b**) nanoemulsion.

DLS analysis gives estimates of the average hydrodynamic size of the particles. This size is usually slightly bigger than the actual physical size because the measurement includes the solvent layer around the particles [42]. The DLS analysis showed the mean diameters of nanoemulsions prepared from *C. cinerariaefolium* and *O. biennis* extracts were 262.5 nm (Figure 2a) and 212.8 nm (Figure 2b). These sizes are in the range expected for liquid nanoparticles. The PDI of the *C. cinerariaefolium* nanoemulsion was 0.493, while that of the *O. biennis* nanoemulsion was 0.267. The lower value of PDI for *O. biennis* (closer to zero) indicated a more homogeneous size distribution than the more stable emulsion of *C. cinerariaefolium*. These results are consistent with the uniformity of the prepared droplets [43], i.e., a uniform droplet size distribution of the nanoemulsion.

#### 3.1.3. Zeta Potential Analysis

**Figure 3:**
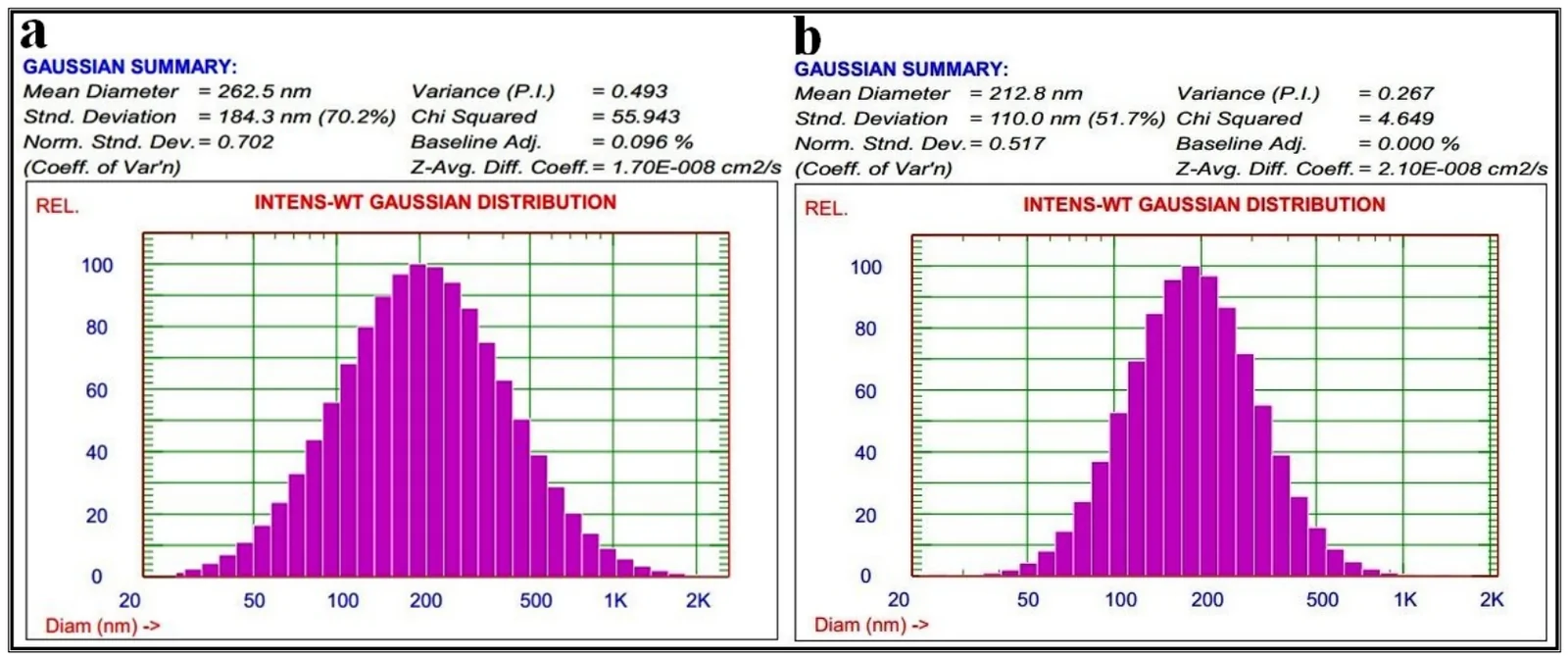
Zeta potential of *Chrysanthemum cinerariaefolium* (**a**) and *Oenothera biennis* (**b**) nanoemulsion.

The observed patterns of zeta potential (Figure 3) confirmed successful formation of nanoemulsions for both extracts (Figure 3a). The power spectrum of the nanoemulsion of *C. cinerariaefolium* showed two different peaks. This means that there is a mixture of particles or complex movements in the emulsion, which is in accordance with the higher value of the PDI. The *O. biennis* nanoemulsion (Figure 3b) exhibited a single power peak, more intense and nearer to zero Hertz, indicating greater homogeneity and electrostatic stability. This is in agreement with the low PDI value, indicating a monodisperse size distribution.

#### 3.1.4. Transmission Electron Microscopy (TEM)

**Figure 4:**
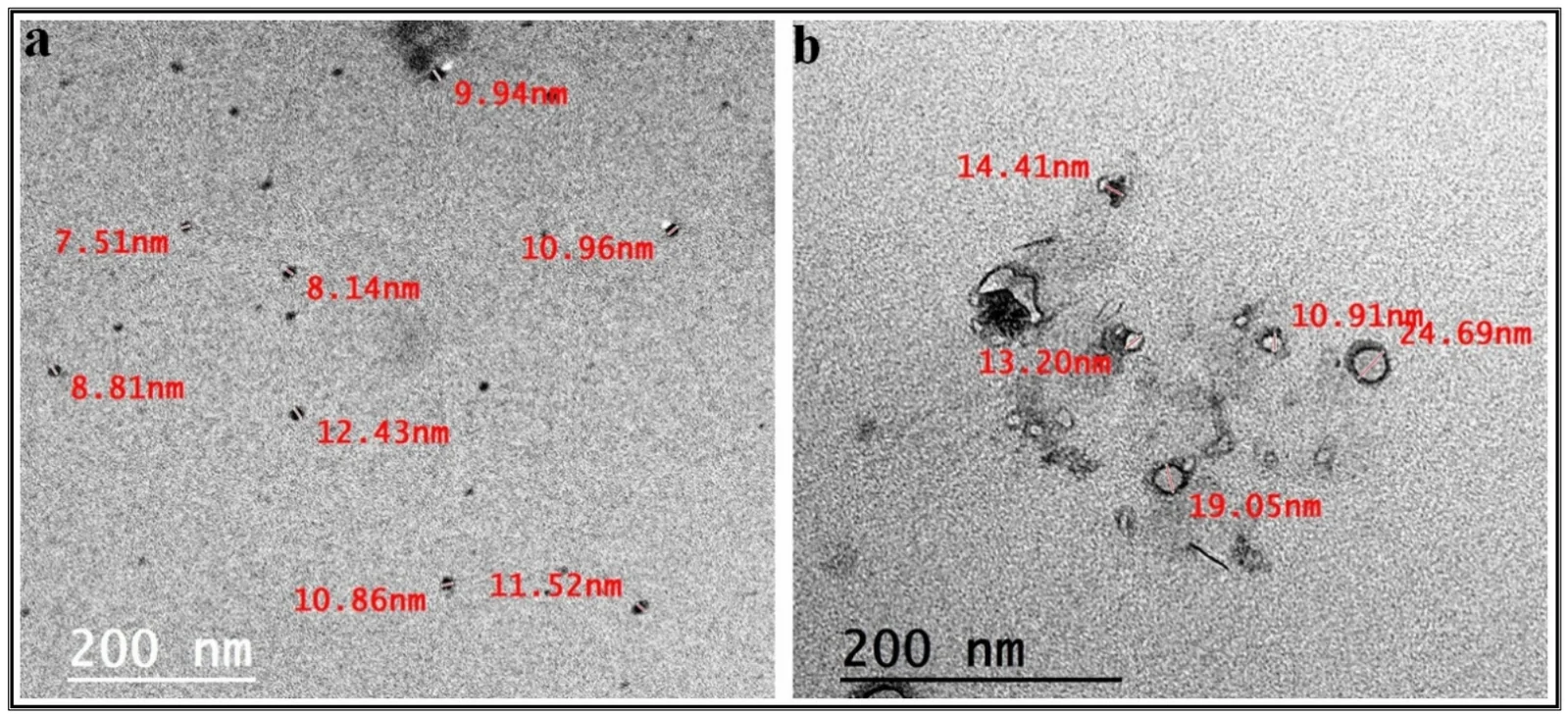
Transmission Electron Microscope of *Chrysanthemum cinerariaefolium* (**a**) and *Oenothera biennis* (**b**) nanoemulsion.

The TEM image (Figure 4a) revealed spherical nanoparticles of *C. cinerariaefolium* with an average width ranging from around 10 nm (e.g., 7.51 to 10.96 nm). This actual size is significantly smaller than the hydrodynamic size estimated by DLS (262.5 nm), confirming the phenomenon of hydrodynamic size measurement in DLS. The TEM image (Figure 4b) of *O. biennis* showed spherical and well-organized nanoparticles with an average width of about 14–19 nm (e.g., 14.41 nm, 19.05 nm, etc.) and some larger particles (24.69 nm) with a regular spherical shape, typical for nanoemulsions [44]. The difference between the DLS sizes (262.5 nm and 212.8 nm) and the TEM sizes (about 10–17 nm) shows the difference between the hydrodynamic size (DLS), which includes the solvent layer and dispersion volume, and the physical/core size (TEM) of the particle in the dry state. The very small-sized TEM (nano-range) confirms the success of the nanoemulsification technique.

### 3.2. Effects of Plant Extracts on Culex pipiens

The flower extracts of *Chrysanthemum cinerariaefolium* and *Oenothera biennis* showed significant larvicidal activity against *Cx. pipiens* larvae. The results showed that *C. cinerariaefolium* exhibited more potent larvicidal activity than *O. biennis*. Nanoemulsion was the best treatment. The larvicidal potency of all treatments was nanoemulsion > methanolic extract > hexane extract > aqueous extract. The larvicidal activity was found to be directly proportional, and exposure times (48 h) resulted in a significant rise in larval mortality when compared to lower concentrations and shorter exposure times. Larval mortality was significantly higher at higher doses and longer exposure time (48 h) than at lower concentrations and shorter exposure time (24 h) (Table 1, Table 2, Table 3 and Table 4).

The crude extracts of *C. cinerariaefolium* showed larval toxicity, and the methanolic extract was most toxic, with 76.00% mortality at 250 ppm and 100% larval mortality at 500 and 1000 ppm. The hexane extract gave slightly less mortality (85.33% at 500 ppm), and the aqueous extract was the least effective, giving approximately 74.67% mortality at the highest concentration. The nanoemulsion formulation of *C. cinerariaefolium* was the most potent insecticidal agent, causing 100% mortality at 250 ppm within 24 h post-treatment (PT). *Oenothera biennis* exhibited similar 24 h PT; the methanolic extract was the most toxic of the crude extracts, causing 80.00% mortality at 500 ppm and complete larval mortality (100%) at 1000 ppm. The hexane extract gave less mortality (85.33% at 1000 ppm). The aqueous extract was least effective, with about 68.00% mortality at the highest concentration. Again, the nanoemulsion showed the most potent effect with complete larval mortality at 250 ppm (Table 1).

After 48 h PT, the mortality percentages further increased across all treatments, confirming the time-dependent nature of toxicity. Both methanolic and hexane extracts of *C. cinerariaefolium* reached full larval mortality at 500 ppm, while the aqueous extract rose to 74.67% at the same concentration. The nanoemulsion maintained its superior performance, sustaining complete mortality even at lower concentrations (125 ppm). By 48 h PT, all *O. biennis* extracts exhibited enhanced efficacy; the methanolic and hexane extracts achieved 100% mortality at 500 ppm, and the aqueous extract increased to approximately 80% at 1000 ppm. The nanoemulsion formulation sustained the highest toxicity level, with 100% mortality recorded at 250 ppm within 48 h (Table 2). The positive control Temephos caused 82.7% and 100% mortality after 24 and 48 h, respectively. Notably, the *C. cinerariaefolium* nanoemulsion in the present study achieved 100% mortality at 250 ppm within only 24 h, demonstrating comparable or superior efficacy.

**Figure 5:**
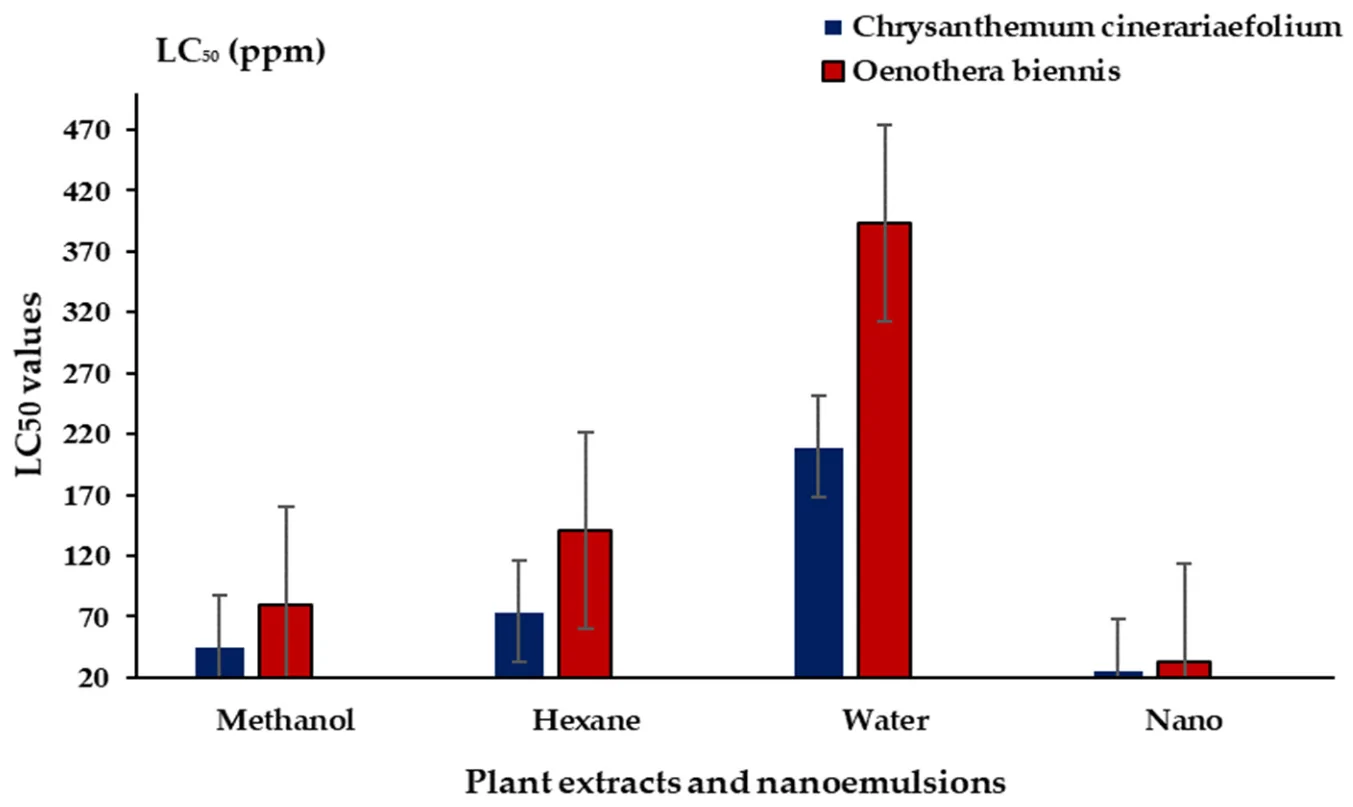
LC_50_ (ppm) values of *Chrysanthemum cinerariaefolium* and *Oenothera biennis* nano extracts on *Cx. pipiens* mortality, 48 h post-treatment.

The probit analysis of *C. cinerariaefolium* and *O. biennis* extracts and nanoemulsions against *Cx. pipiens* larvae revealed distinct variations in larvicidal potency depending on the solvent and exposure time (Table 3 and Table 4). Consistent with these findings, the LC_50_ values confirmed the superior activity of nanoemulsion formulations. For *C. cinerariaefolium*, the 24 h PT LC_50_ values decreased from 557.56 ppm (water), 163.31 ppm (hexane), and 94.81 ppm (methanol) to only 43.89 ppm for the nanoemulsion. After 48 h PT, the LC_50_ further dropped to 26.63 ppm, indicating time-dependent toxicity enhancement (Table 3 and Figure 5). Similarly, *O. biennis* extracts demonstrated higher LC_50_ values for crude extracts (178.43 ppm for methanol, 324.67 ppm for hexane, and 673.26 ppm for water), while the nanoemulsion recorded a markedly lower LC_50_ of 58.28 ppm after 24 h, which decreased to 31.89 ppm after 48 h. These results highlight that nanoformulations significantly improved larvicidal efficacy, requiring smaller doses to achieve comparable or superior mortality to conventional extracts (Table 4 and Figure 5).

### 3.3. Biochemical and Oxidative Stress of Treated Mosquito Larvae

The biochemical study focused on the most active treatments at their LC_50_ concentrations, namely the methanolic extracts and nanoemulsions of the tested botanical insecticides. Exposure of mosquito larvae to the LC_50_ of methanolic extracts and nanoemulsions of *C. cinerariaefolium* and *O. biennis* induced pronounced alterations in esterase enzyme activities (Table 5). A marked suppression was recorded in acetylcholinesterase (AChE), α-esterase, and *β*-esterase activities, particularly in larvae treated with *C. cinerariaefolium* formulations. The nanoemulsion of *C. cinerariaefolium* exhibited the strongest inhibitory effects (4.24, 1.02, and 1.37 units, respectively) compared with the control (9.44, 2.27, and 3.05 units, respectively). The methanolic extract of *C. cinerariaefolium* also significantly reduced enzyme activities (5.25, 1.26, and 1.70 units, respectively). In contrast, *O. biennis* showed minor variations relative to the control, with its nanoemulsion producing moderate reductions (7.47, 1.80, and 2.42 units, respectively).

A similar pattern was observed in infection-related enzymes, where *C. cinerariaefolium* extract and its nanoemulsion caused pronounced inhibitory effects on gamma-aminobutyric acid transaminase (GABA-T), amylase, and lipase activities (Table 6). The nanoemulsion of *C. cinerariaefolium* yielded the lowest activity values (1.96, 8.67, and 0.85 units/g tissue, respectively) compared with the control (4.50, 19.30, and 1.90 units/g tissue), followed by the methanolic extract (2.52, 10.73, and 1.05 units/g tissue, respectively). Meanwhile, *O. biennis* and its nanoemulsion induced only slight to moderate decreases, maintaining values close to the control group (3.56, 15.28, and 1.50 units/g tissue, respectively).

The antioxidant defense system of mosquito larvae was markedly affected following treatment with *C. cinerariaefolium* and *O. biennis* extracts and nanoemulsions (Table 7). The *C. cinerariaefolium* nanoemulsion produced the most pronounced oxidative imbalance, reflected by decreased SOD, GST, CAT, and GSH activities (1.09, 25.35, 1.64, and 0.74 units, respectively) and markedly elevated LPO and TPC levels (12.16 and 15.09 nmol/mg protein, respectively) compared with the control (2.42, 56.41, 3.65, and 1.65 units, respectively) and (3.50 and 5.42 nmol/mg protein). The methanolic extract of *C. cinerariaefolium* also triggered substantial reductions in antioxidant enzymes, though to a lesser extent (1.35, 31.37, 2.03, 0.92 units, respectively) and (6.16 and 9.56 nmol/mg protein). Conversely, *O. biennis* exhibited mild alterations, indicating limited oxidative disturbance relative to the control. These findings highlight the potent biochemical and oxidative impacts of *C. cinerariaefolium* formulations, supporting their potential as eco-friendly botanical larvicides.

### 3.4. Phytochemical Investigation

#### 3.4.1. GC/MS Identification of the Volatile Metabolites from *Chrysanthemum cinerariaefolium* Flowers *n*-Hexane Extract

The *n*-hexane flower extract of *C. cinerariaefolium* was subjected to GC/MS analysis in order to reveal its volatile phytochemical profile. Forty-nine components were identified (64.52% identification) and listed in Table 8. The identified compounds belonged mainly to hydrocarbons, oxygenated hydrocarbons, and triterpenoids, in addition to sesquiterpenoids, fatty acids, and their esters. Among the major peaks, hexatriacontane (10.81%), tetracontane (5.74%), hexanedioic acid, bis(2-ethylhexyl) ester (5.25%), tetracosane (4.06%), eudesm-4(14)-en-11-ol (3.34%), (+)-ledene (2.90%), and α-guaiene (2.05%) were detected.

#### 3.4.2. GC/MS Identification of the Volatile Metabolites from *Oenothera biennis* Flowers *n*-Hexane Extract

GC/MS analysis was utilized for the identification of the volatile metabolites from *O. biennis n*-hexane flower extract. As detailed in Table 9, the % identification was recorded as 78.03%. The main identified classes were hydrocarbons and oxygenated hydrocarbons in the form of sesqui-, di-, and triterpenoids. Tetracontane (20.74%), hexatriacontane (13.79%), heneicosane (7.11%), eicosane (4.42%), and tetradecanal (3.94%) were listed as the major identified peaks.

#### 3.4.3. UPLC/MS Tentative Identification of Metabolites from Methanol, Ethyl Acetate and Aqueous Extracts of *Chrysanthemum cinerariaefolium* Flowers

**Figure 6:**
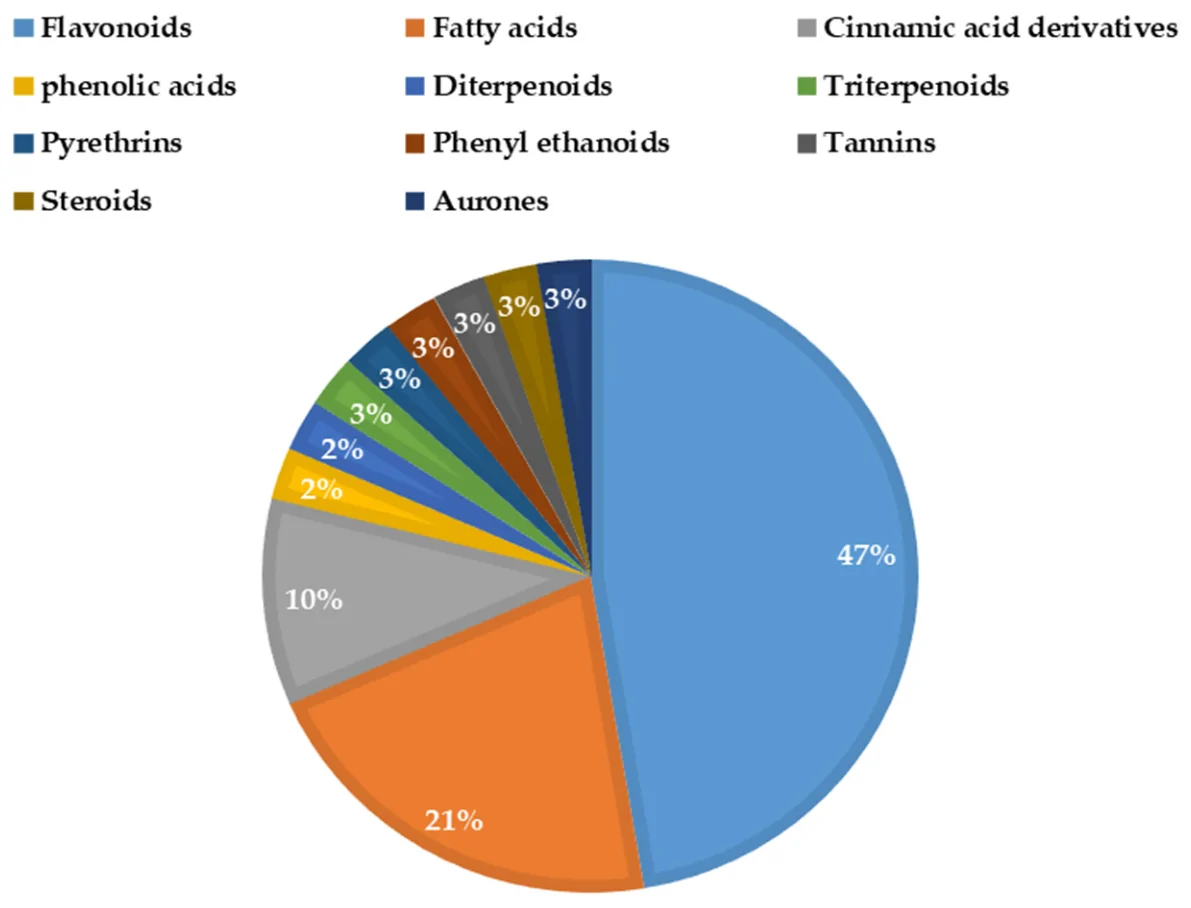
The tentatively identified metabolites from the flower extracts of *Chrysanthemum cinerariaefolium*.

Different extracts, namely methanol (CW-Me), ethyl acetate (CW-Eth. Ac.), and aqueous (CW-Aq.), were prepared from the flowers of *Chrysanthemum cinerariaefolium*. Their phytochemical profiles were investigated and compared using UPLC/MS analysis in ESI negative and positive modes. Thirty-eight metabolites were tentatively identified and listed in Table S1, where flavonoids and fatty acids were the predominant classes. In addition, cinnamic acid derivatives, phenolic acids, diterpenoids, triterpenoids, pyrethrins, phenyl ethanoids, steroids, aurones, and tannins were also detected (Figure 6).

**Figure 7:**
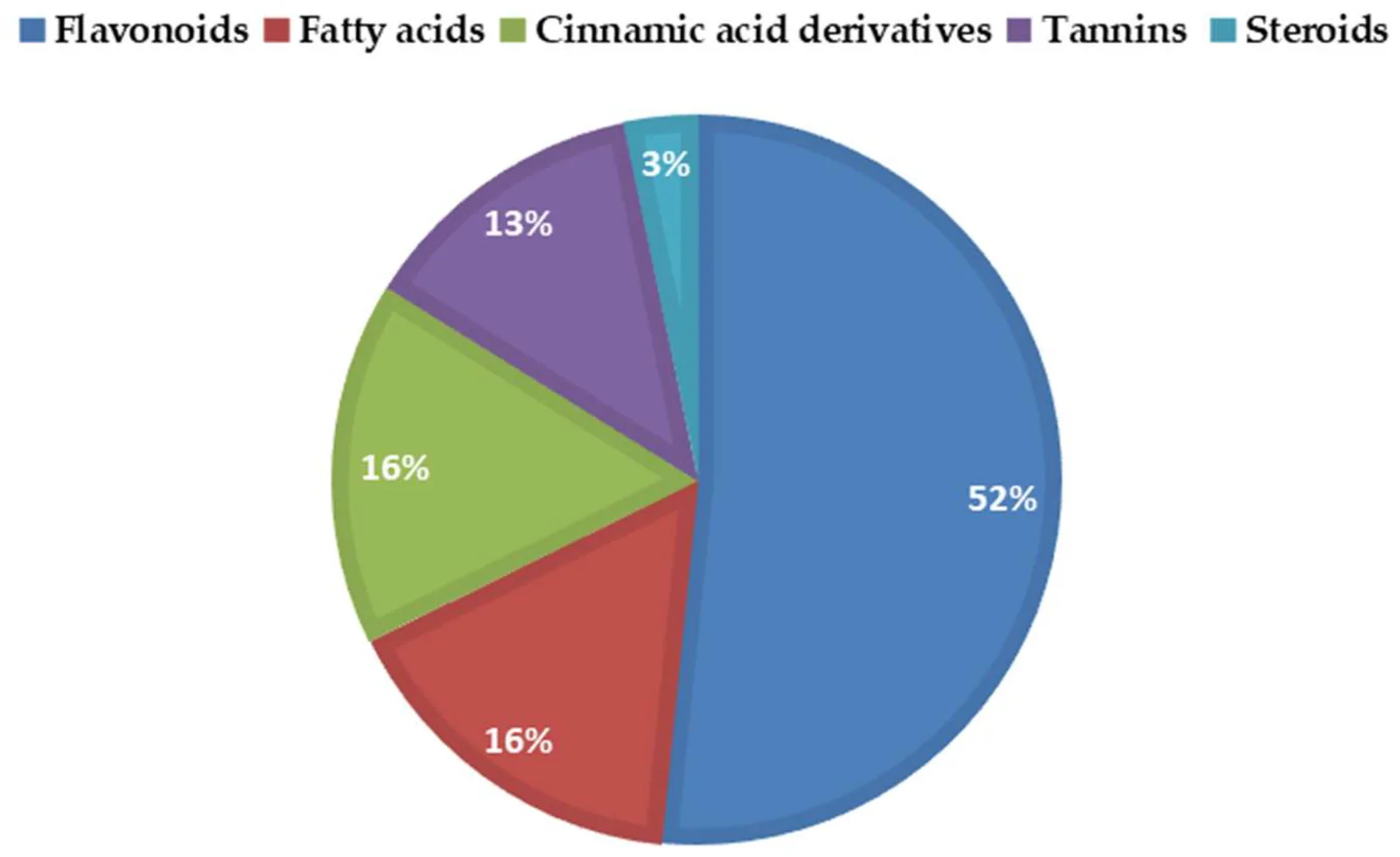
The tentatively identified metabolites from the flower extracts of *Oenothera biennis*.

Eighteen flavonoids were tentatively identified and listed in Table S1 and Figure 7. An abundant peak appeared at *m*/*z* 377/381 with % composition of 8.25 (32.51) from the methanol extract (CW-Me) and 5.19 (21.37) from the ethyl acetate extract (CW-Eth. Ac.). This peak was tentatively assigned to *tetra*-hydroxy-*tri*-methoxy-*di*-hydroxyflavone [45]. Similarly, other flavones and flavanones were also detected, *viz*. *tetra*-hydroxy-flavanone [46], *tetra*-hydroxy-*di*-methoxyflavone [47] and hydroxy-methoxy-methylenedioxy-dimethyl homoisoflavanone [48], which presented deprotonated peaks at *m*/*z* 287, *m*/*z* 345 and *m*/*z* 355, respectively.

Moreover, certain aglycones such as kaempferol, apigenin, quercetin, isorhamnetin, acacetin, naringenin, chrysoeriol and luteolin were reported from the genus *Chrysanthemum* and were also detected herein from the three flower extracts. Among such flavonoids, kaempferol-galloyl-hexoside (*m*/*z* 599) [49], apigenin-hexoside (*m*/*z* 431) [47,50,51], quercetin-rutinoside (*m*/*z* 609/771) [51,52], luteolin-hexuronyl-hexoside (*m*/*z* 623/625) [53], isorhamnetin-hexoside (*m*/*z* 477) [51], luteolin-hexoside (*m*/*z* 447) [50,51], chrysoeriol (*m*/*z* 299) [52], acacetin-hexoside (*m*/*z* 445) [54,55], acacetin-malonyl-hexoside (*m*/*z* 531) [45], apigenin (*m*/*z* 269/277) [56] and naringenin (*m*/*z* 271) [47,52] were tentatively identified, and their respective molecular formulas and % compositions were detailed in Table S1.

Fatty acids came second after flavonoids in abundance; eight fatty acids, including one oxylipin, were tentatively identified. Two deprotonated peaks were detected at *m*/*z* 309 and *m*/*z* 277, which were tentatively identified as linolenic acid-hydroperoxide [57] and linolenic acid [58]. In addition, several other peaks were also observed and were tentatively identified and listed as fatty acids such as *trans*-vaccenic acid (*m*/*z* 281, reported from the genus *Chrysanthemum*) [47], *di*-hydroxy-*oxo*-octadecenoic acid (*m*/*z* 327, 3.89% CW-Me, 5.51% CW-Eth. Ac., reported from the genus *Chrysanthemum*) [47], *oxo*-octadecadienoic acid (*m*/*z* 293, 12.55% CW-Me, 21.26% CW-Eth. Ac.) [59], hexopyranosyloxy-hydroxy-propyl ester octadecatrienoic acid (*m*/*z* 559) [47], hydroxy-octadecadienoic acid (*m*/*z* 295/279, 7.33% CW-Me, 12.66% CW-Eth. Ac., reported from the genus *Chrysanthemum*) [47] and octadecadienoic acid (*m*/*z* 279) [58].

Moreover, certain cinnamic acid derivatives were recorded as chlorogenic acid [47,50,51], *di*-caffeoyl-quinic acid (7% CW-Me) [60], *tri*-methoxy-cinnamoyl-quinic acid [61] and caffeoyl quinate shikimate derivative [62] with their respective deprotonated peaks detected at *m*/*z* 353, *m*/*z* 515, *m*/*z* 411 and *m*/*z* 509. Likewise, caffeic acid appeared in ESI −negative mode at *m*/*z* 179 only from CW-Aq. (2.53%) [51]. Other metabolites appeared as one compound from each phytochemical class, and they can be detailed as cafestol, which is a diterpenoid reported from the genus *Chrysanthemum* and showed its peak at *m*/*z* 317 (ESI + ve mode) [47]. Similarly, other metabolites were tentatively identified as *mono*-hydroxyl-*mono*-methoxy-ursolic acid (triterpenoid, *m*/*z* 501/503) [45,63], pyrethrin I (pyrethrin, *m*/*z* 329, 4.92% CW-Eth. Ac., 2.06% CW-Aq.) [64], phenethyl sophoroside (phenyl ethanoid, *m*/*z* 439) [47], proanthocyanidin dimer (tannin, *m*/*z* 561), α-hydroxy-deoxy-cholic acid (steroid, *m*/*z* 391) [65] and Bracteatin (aurone, *m*/*z* 303) [47].

#### 3.4.4. UPLC/MS Tentative Identification of Metabolites from Methanol, Ethyl Acetate and Aqueous Extracts of *Oenothera biennis* Flowers

Three extracts, namely methanol (OBF-Me), ethyl acetate (OBF-Eth. Ac.), and aqueous (OBF-Aq.), were investigated through UPLC/MS analysis, and thirty-one metabolites were tentatively identified from the three extracts; the most abundant class was that of flavonoids, followed by fatty acids, cinnamic acid derivatives, and tannins in addition to steroids.

Flavonoids were recorded as the most abundant category of identified metabolites from *Oenothera biennis* flower extracts, accounting for almost half of the number of identified metabolites as listed in Table S2. Apigenin (*m*/*z* 269, 8.91% OBF-Aq.), luteolin (*m*/*z* 285/287) [66], naringenin (*m*/*z* 271/273, major peak at 39.83% OBF-Me, 38.52% OBF-Eth. Ac.) [66] and chrysoeriol (*m*/*z* 299, 3.53% OBF-Me, 4.48% OBF-Eth. Ac.) [52] were among the flavonoid aglycones that were tentatively identified herein and previously recorded from the genus *Oenthera*.

In addition to the aforementioned aglycones, several other flavonoid glycosides, flavones, flavanones, and other flavonoid derivatives were tentatively identified, such as *tetra*-hydroxy-*tri*-methoxy-*di*-hydroxyflavone (*m*/*z* 377/381) [45], quercetin-pentoside (*m*/*z* 447, 4.20% OBF-Aq. only) [67], apigetrin (*m*/*z* 431) [68], quercitrin-*di*-hexoside (*m*/*z* 771) [69,70], quercetin hexuronyl-hexoside (*m*/*z* 639) [71], rutin (*m*/*z* 609) [66], isorhamnetin-rutinoside (*m*/*z* 623/625, 6.55% OBF-Me, 3.15% OBF-Eth. Ac., 3.24% OBF-Aq.) [72,73], kaempferol-hexuronide (*m*/*z* 461) [71], rhamnetin-hexuronide (*m*/*z* 491, 4.43% OBF-Me) [74], quercetin-dimethyl ether (*m*/*z* 329/331) [75], *tetra*-hydroxy-flavanone (*m*/*z* 287) [46] and syringetin-hexoside (*m*/*z* 507) [76].

In addition to that, five fatty acids were tentatively identified as *di*-homo-*γ*-linolenic acid, *oxo*-*di*-hydroxy-octadecenoic acid [73,77], *oxo*-octadecadienoic acid (6.49% OBF-Me, 14.97% OBBF-Eth. Ac.) [59], hydroxy-octadecadienoic acid (5.08% OBF-Me, 7.38% OBF-Eth. Ac.) [47] and linoleic acid [71] with their deprotonated/protonated molecular ion peaks detected at *m*/*z* 307, *m*/*z* 327, *m*/*z* 293/317, *m*/*z* 295/279 and *m*/*z* 279, respectively. Likewise, several caffeoyl derivatives were tentatively detected and may be detailed as caffeoyl hexoside (*m*/*z* 341) [78], chlorogenic acid (*m*/*z* 353/355) [79], *di*-hydro-caffeic acid (*m*/*z* 181, 5.43% OBF-Aq. only) [80], *di*-caffeoyl-quinic acid (*m*/*z* 515/565) [60] and caffeic acid pentoside (*m*/*z* 311) [71].

Besides, four tannin peaks were recorded at *m*/*z* 467, *m*/*z* 451, *m*/*z* 305 and *m*/*z* 357, which were tentatively assigned to catechin-caffeic adduct [81], catechin-hexoside [82], (*epi*) gallocatechin (3.15% OBF-Aq. only) [78,83] and *tetra*-methyl-ellagic acid [71], respectively.

#### 3.4.5. Multivariate Data Analysis for the Identified Metabolites

**Figure 8:**
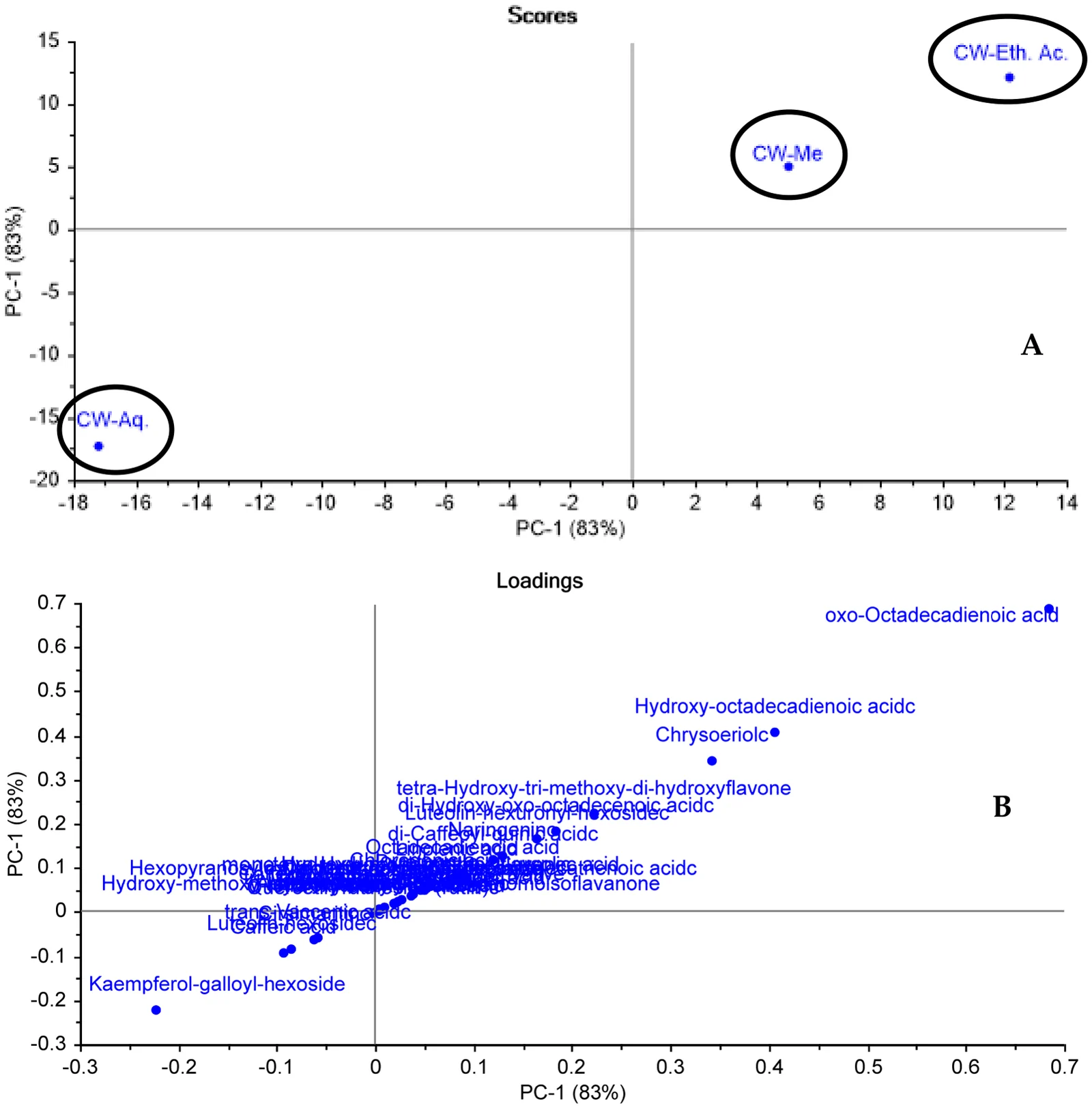
(**A**) Score plot of PC1 *versus* PC2 of the UPLC/MS identified metabolites from *Chrysanthemum cinerariaefolium* methanol, ethyl acetate, and aqueous flower extracts (area % as a variable). (**B**) Loading plot for PC1 and PC2 contributing metabolites and their assignments (area % as a variable). CW-Me: *C. cinerariaefolium* methanol extract, CW-Eth. Ac.: *C. cinerariaefolium* ethyl acetate extract and CW-Aq.: *C. cinerariaefolium* aqueous extract.

**Figure 9:**
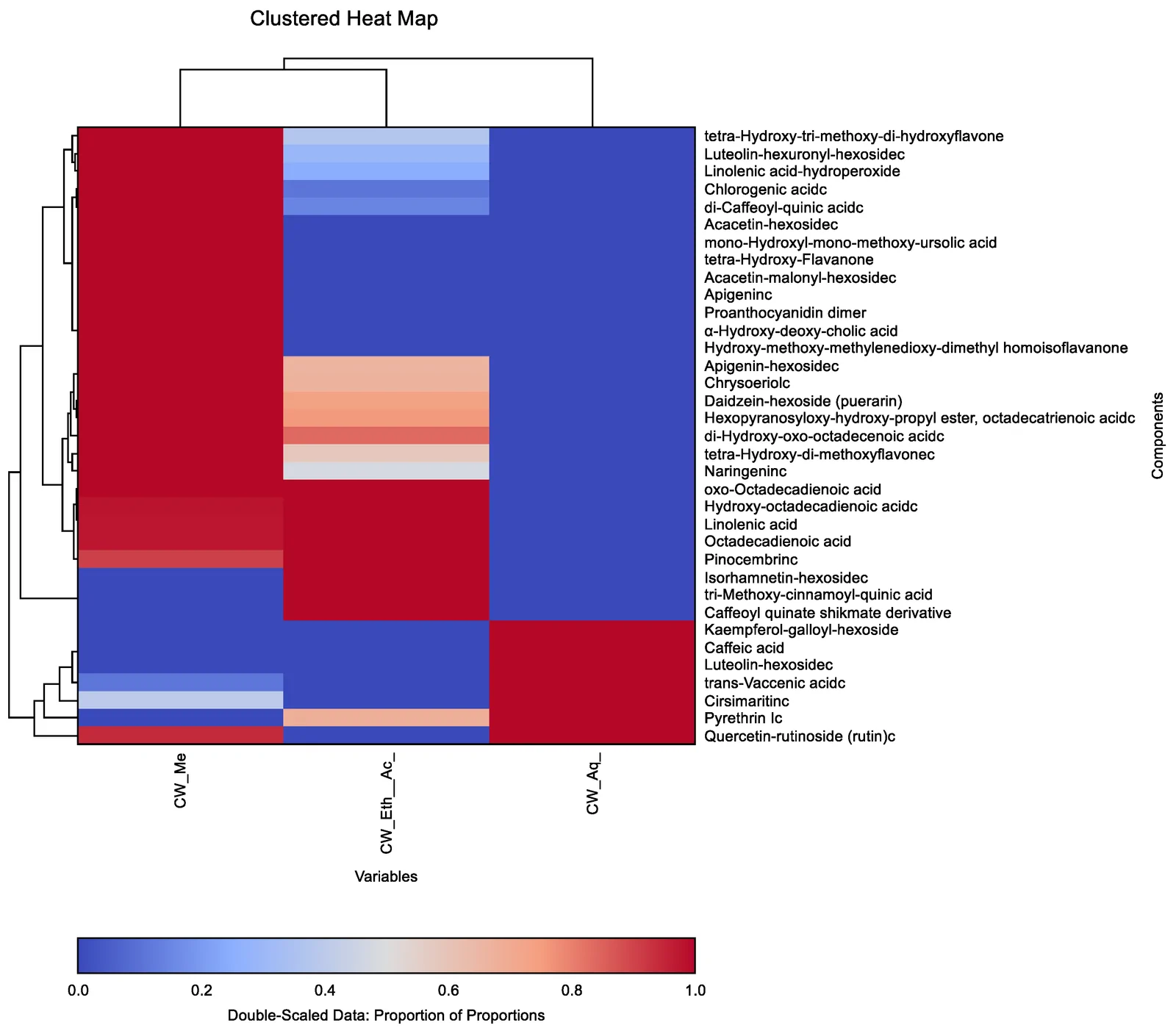
Clustered heat map showing the tentatively identified metabolites (UPLC/MS, negative mode) from *Chrysanthemum cinerariaefolium* flower extracts. A heat map was constructed using Euclidean distance and the unweighted group method. (CW-Me: *C. cinerariaefolium* methanol extract, CW-Eth. Ac.: *C. cinerariaefolium* ethyl acetate extract and CW-Aq.: *C. cinerariaefolium* aqueous extract).

Principal component analysis (PCA), an unsupervised multivariate data analysis tool, and a hierarchical cluster heat map were applied herein in order to discriminate the tentatively identified metabolites from the UPLC/MS data in negative ion mode. The tentatively identified metabolites from each plant extract were separately analyzed using PCA and a clustered heat map. As shown in Figure 8A, the PCA score plot for *Chrysanthemum cinerariaefolium* extracts represented three distinct clusters, with one located in the left lower quadrant for CW-Aq. and two other nearby clusters located in the same right upper quadrant for CW-Me and CW-Eth. Ac.. This clustering pattern could be explained by the PCA loading plot (Figure 8B) and the unique phytochemical composition of the CW-Aq. extract compared to the other two, as certain metabolites, *viz*. kaempferol-galloyl-hexoside, were abundant only in CW-Aq., thus clustering this extract in a separate quadrant. This same clustering pattern also appears in the clustered heat map constructed in Figure 9, where the areas in red represent the metabolites with the highest % composition and those in blue represent the lowest % composition.

**Figure 10:**
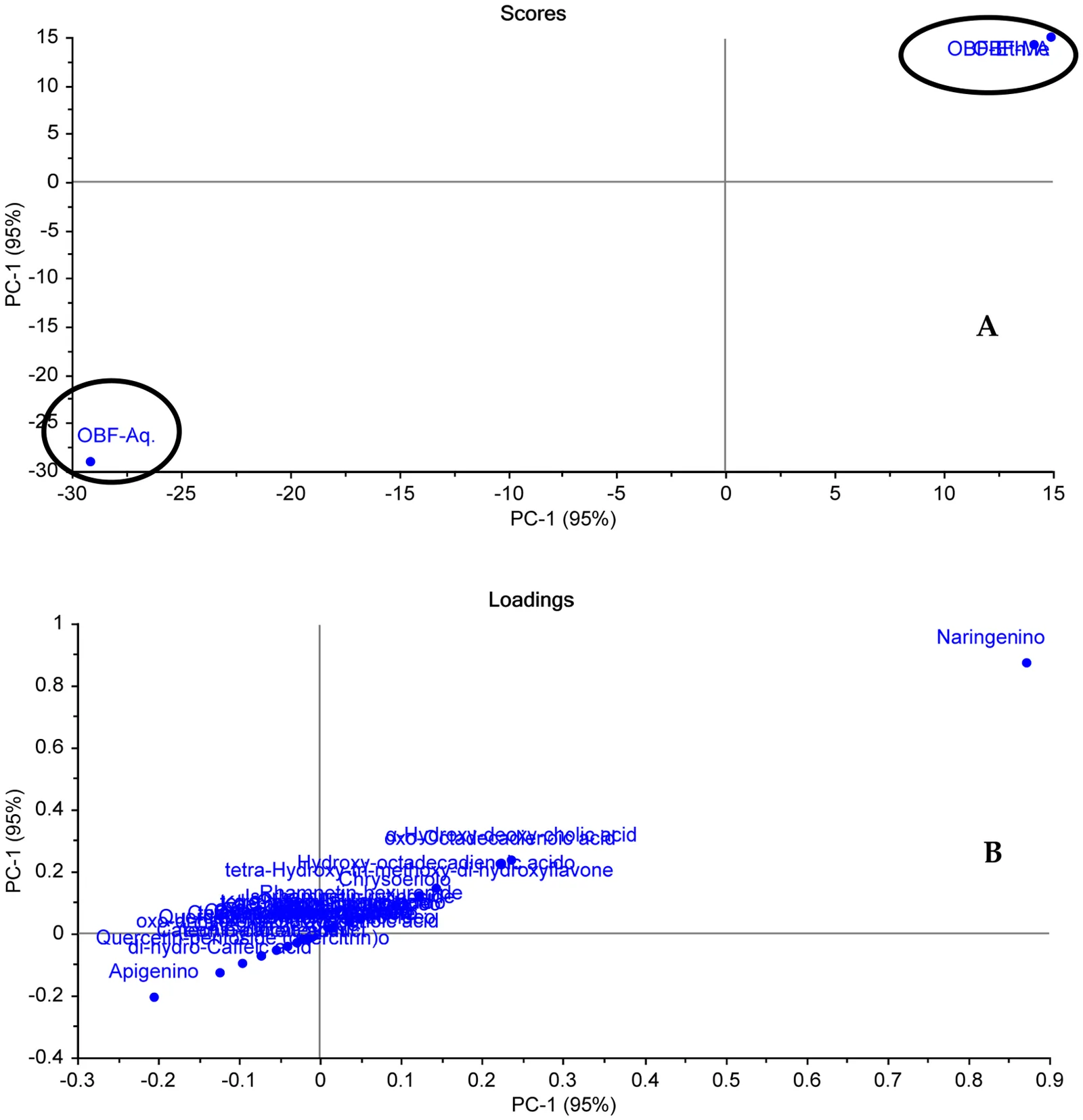
(**A**) Score plot of PC1 *versus* PC2 of the UPLC/MS identified metabolites from *Oenothera biennis* methanol, ethyl acetate, and aqueous flower extracts (area % as a variable). (**B**) Loading plot for PC1 and PC2 contributing metabolites and their assignments (area % as a variable). OBF-Me: *O. biennis* methanol extract, OBF-Eth. Ac.: *O. biennis* ethyl acetate extract and OBF-Aq.: *O. biennis* aqueous extract.

**Figure 11:**
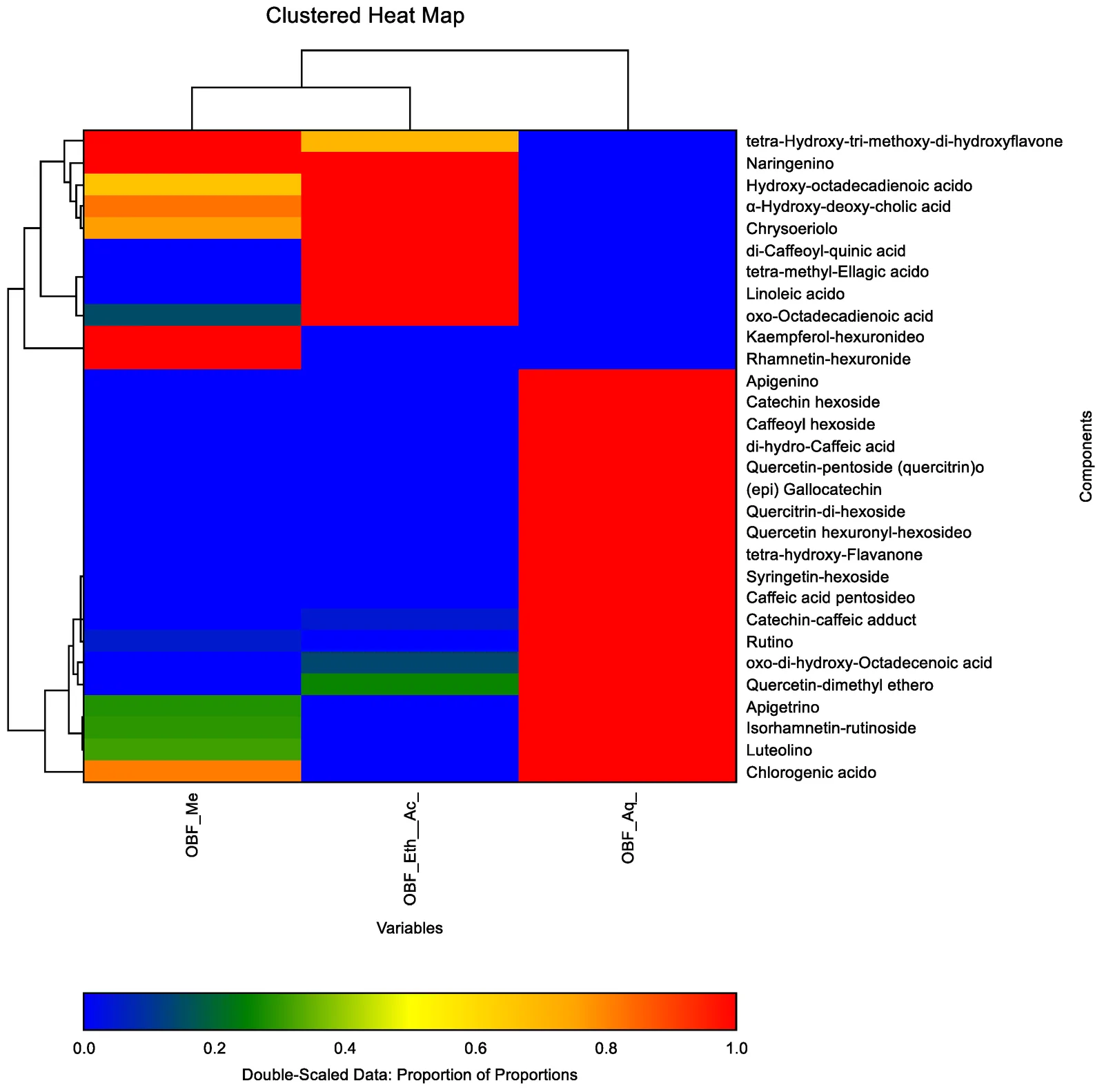
Clustered heat map showing the tentatively identified metabolites (UPLC/MS, negative mode) from *Oenothera biennis* flower extracts. A heat map was constructed using Euclidean distance and the unweighted group method. (OBF-Me: *O. biennis* methanol extract, OBF-Eth. Ac.: *O. biennis* ethyl acetate extract and OBF-Aq.: *O. biennis* aqueous extract).

Similarly, the UPLC/MS tentatively identified metabolites from *Oenothera biennis* flower extracts were discriminated using PCA and a clustered heat map. The PCA score plot was illustrated in Figure 10A, where two distinct and separate clusters were recorded. The first was located in the left lower quadrant for OBF-Aq. while the other cluster appeared in the right upper quadrant, showing an overlap between the OBF-Me and OBF-Eth. Ac. extracts. This clustering pattern could be clarified by the PCA loading plot (Figure 10B), where the unique and different phytochemical composition was displayed by OBF-Aq. such as the apigenin aglycone, which appeared as the sole abundant metabolite in this extract. In addition, the overlapping cluster between OBF-Me and OBF-Eth. Ac. may be explained by their nearly similar metabolite makeup, which was revealed in the PCA loading plot. Moreover, this clustering pattern was also recorded in the clustered heat map illustrated in Figure 11, where the color pattern ranged from blue for the metabolites with low % composition to red for those with high % composition.

## 4. Discussion

Mosquitoes are persistent pests, and the diseases they transmit continue to threaten global health. *Culex pipiens* is a common species that spreads serious diseases in both people and animals. Controlling mosquitoes effectively is vital for public health and for maintaining the integrity of ecosystems and food chains [84,85]. Despite advances in entomology, reliance on chemical insecticides remains high, largely due to the rapid emergence of resistance. Furthermore, the ecological hazards of synthetic insecticides, including their persistence and toxicity, have prompted strict regulations, which aim to mitigate their harmful effects on non-target species and ecosystems. These challenges have driven the search for natural, sustainable, and eco-friendly alternatives for pest management [10].

Natural products provide dual advantages: they diminish the transmission of mosquito-borne pathogens while reducing environmental hazards linked to chemicals [86]. Using eco-friendly substances also helps prevent resistance and protects ecosystems [87,88]. Numerous natural substances, such as medicinal plant extracts and essential oils, have demonstrated significant larvicidal efficacy against *Cx. pipiens* larvae [89,90].

This study evaluated the larvicidal efficacy of *C. cinerariaefolium* and *O. biennis* extracts, along with their nanoemulsion formulations. All tested extracts induced larval mortality, with efficacy varying according to solvent type and concentration. LC_50_ values indicated that *C. cinerariaefolium* was more potent than *O. biennis*. After 48 h, the *C. cinerariaefolium* nanoemulsion exhibited the highest toxicity (LC_50_ = 26.63 ppm), followed by methanol (44.46 ppm), hexane (73.90 ppm), and aqueous extracts (209.99 ppm). For *O. biennis*, LC_50_ values were 31.89 ppm for the nanoemulsion, 80.15 ppm for methanol, 140.69 ppm for hexane, and 393.22 ppm for aqueous extracts.

Our results match previous studies highlighting the potent larvicidal activity of *C. cinerariaefolium* against *Anopheles* gambiae [91]. Among various solvents, DCM and ethanol flower extracts exhibited the highest potency, while aqueous extracts demonstrated lower activity, indicating limited extraction of active compounds in water. The exceptional insecticidal potency of *C. cinerariaefolium* is attributed to pyrethrin esters, resulting from the esterification of chrysanthemic and pyrethric acids. These pyrethrins are exclusively found in this species [92]. Our findings are also supported by studies on *C. morifolium*, where methanolic and DCM extracts showed strong toxicity against *Aedes aegypti* due to triterpenoids, fatty acids, and flavonoids [93,94]. These metabolites target pests through various mechanisms, such as acting on acetylcholine receptors or sodium channels [95].

Consistent with our findings, Ambrin et al. [96] reported that ethyl acetate extracts of Oenothera biennis exhibited strong activity against stored product pests. Furthermore, *O. biennis* is rich in polyphenolic compounds and antioxidants [97]. Previous research has also demonstrated its antimicrobial properties against various pathogens like *S. aureus* and *P. aeruginosa* [98,99]. Recent investigations into its roots identified oenotheralanosterol B as a potent bioactive agent [100].

The current study revealed that larvicidal potency followed the order: nanoemulsion > methanolic extract > hexane extract > aqueous extract. The efficiency of phytochemical extraction is strongly influenced by the polarity of the solvent. Non-polar solvents like n-hexane are effective for lipophilic compounds (terpenoids, fatty acids), while polar solvents like methanol and water are better for phenolics and glycosides. These differences markedly affect efficacy; for instance, Bosly [101] found that polar fractions of Thuja orientalis were more effective against *Cx. pipiens*, whereas Farag [102] reported that non-polar extracts of Sesamum indicum yielded greater mortality. This reflects the diverse distribution of active metabolites across different plant species [103].

The significant reduction in LC_50_ values of the nanoemulsion formulations compared to their crude counterparts highlights the superior bio-efficacy of the nano-delivery system. This enhancement is attributed to the reduced droplet size, which increases the surface area and facilitates the penetration of bioactive compounds through the insect’s lipophilic cuticle. Similar findings were reported by Mossa et al. [104] with camphor oil nanoemulsions. The nano-shuttle system prevents the degradation of phytochemicals and ensures higher concentrations at target sites.

The significant reduction of LC_50_ values of nanoemulsion formulations as compared to crude counterparts is indicative of the superior bioefficacy of the nanodelivery system. A common larvicide, Temephos, normally kills very well at very low concentrations (ppm). We previously showed that the recommended field concentration resulted in 86.7 and 100% mortality at 24 and 48 h, respectively [25]. Interestingly, the nanoemulsion of *C. cinerariaefolium* in the present study showed 100% larval mortality within 24 h at the concentration of 250 ppm. This concentration is higher than that of Temephos, but note that botanical nanoemulsions have specific advantages such as biodegradability, lower environmental persistence, lower toxicity to non-target organisms, and a multi-modal mode of action that reduces the risk of resistance development. The present results reveal the potential use of plant-based nano-formulations as a promising eco-friendly alternative for integrated vector management. In alignment with our results, Sugumar et al. [105] observed that botanical nanoemulsions significantly enhanced larvicidal potency. Furthermore, encapsulating bioactive compounds protects them from environmental degradation, ensuring sustained release [106]. The small droplet size provides a larger surface area for interaction with larval membranes, inducing neurotoxicity and disrupting normal functions [107].

The thorough characterization utilizing four distinct methodologies validated the successful synthesis and stability of the nanoemulsions. UV spectroscopy confirmed the integration of bioactive compounds [40]. DLS analysis showed larger hydrodynamic diameters (212.8–262.5 nm) because it includes the surrounding solvent layer and surface metabolites [42]. However, the definitive confirmation was provided by TEM imaging, revealing the physical core size to be approximately 10–19 nm. This difference between DLS and TEM is typical and validates that the core particles are within the desired nano-range [44]. Polydispersity Index (PDI) and Zeta Potential values further confirmed the homogeneity and adequate repulsive forces required for stable colloidal systems [43].

Exposure to *C. cinerariaefolium* methanolic extracts and nanoemulsions at LC_50_ induced significant biochemical disruptions, notably suppressing esterases, neuro-related enzymes (GABA-T), digestive enzymes (amylase, lipase), and antioxidant defenses (SOD, GST, CAT, GSH), while elevating LPO and TPC levels. The insecticidal activity of these extracts is primarily attributed to monoterpenes and fatty acids [108]. These constituents inhibit acetylcholinesterase (AChE) and interact with neurotransmitter receptors, causing paralysis or death [109]. Additionally, fatty acids like palmitoleic and lauric acids possess insecticidal potential [110]. Essential oils and extracts can compromise the integrity of the insect cuticle, facilitating toxin penetration and modulating the expression of key physiological proteins [111]. Using complex mixtures of phytochemicals through nano-formulations helps mitigate resistance development and overcomes the limitation of high volatility, thereby enhancing the effectiveness of insecticides and ensuring longer-lasting pest control [112].

The current study provided comprehensive comparative phytochemical profiling of various extracts from *C. cinerariaefolium* and *O. biennis*. This characterization employed GC-MS analysis for *n*-hexane extracts to identify non-polar volatile constituents, while UPLC-MS/MS was utilized for the more polar methanol, ethyl acetate, and aqueous fractions. The GC-MS analysis of the *n*-hexane extracts revealed shared phytochemical classes between the two species, including hydrocarbons, oxygenated hydrocarbons, sesquiterpenoids, and fatty acids.

In contrast, the metabolites tentatively identified via UPLC-MS/MS were predominantly grouped into flavonoids, cinnamic acid derivatives, and fatty acids as common classes. *Chrysanthemum cinerariaefolium* extracts exhibited a more complex metabolic profile; in addition to the shared classes, they were uniquely characterized by di- and triterpenoids, steroids, aurones, and, most significantly for insecticidal activity, pyrethrins.

These findings align with previous phytochemical investigations that utilize chromatographic and mass spectrometric approaches to reveal the secondary metabolites responsible for biological activities. Due to their wide phytochemical variety and traditional usage, *Chrysanthemum* species (Asteraceae) have been studied extensively. GC-MS investigations of *Chrysanthemum indicum* and related species have identified various terpenoids, phenolic derivatives, and fatty acids with known antibacterial, antioxidant, and anti-inflammatory properties. Furthermore, complementary LC-MS-based metabolomic techniques have highlighted the genus’s complexity, identifying over 100 phytoconstituents such as flavonoids, phenolic acids, and carotenoids, which underscores its promise as a source of pharmacologically active compounds [113,114].

Similarly, members of the genus *Oenothera* (Onagraceae), particularly *O. biennis* and *O. rosea*, have gained attention for their medicinal and nutraceutical values. In this study, GC-MS profiling of *O. biennis* identified several antioxidant-related components, including fatty acids and flavone derivatives, supporting the plant’s capacity for free radical scavenging. Advanced LC-MS metabolomics on *O. rosea* preparations has further identified hundreds of metabolites linked to anti-inflammatory and anticancer properties. These combined results emphasize the necessity of integrating GC-MS and LC-MS methodologies for the thorough characterization of both volatile and non-volatile phytochemicals. Such a comprehensive approach allows for a more profound understanding of the chemical diversity that drives the medicinal and insecticidal applications of these plant extracts [75,115].

It is important to acknowledge the limitations of this study. The larvicidal efficacy was evaluated exclusively under controlled laboratory conditions, which may not fully represent the complex and variable field environments. Furthermore, our investigation did not extend to assessing the residual activity or long-term stability of the nanoemulsions under various environmental conditions (e.g., temperature and sunlight) or their potential toxicity to non-target aquatic organisms. Therefore, while our results are highly promising, future research should focus on field evaluations, formulation optimization for enhanced stability, and comprehensive ecotoxicological risk assessments to confirm the practical applicability and environmental safety of these botanical nanoemulsions as sustainable vector control agents.

## 5. Conclusions

The present study demonstrates that *Chrysanthemum cinerariaefolium* and *Oenothera biennis* extracts, particularly their methanolic and nanoemulsion formulations, exhibit potent larvicidal activity against *Culex pipiens* larvae. *C. cinerariaefolium* exhibited superior efficacy compared to *O. biennis*, characterized by a significant suppression of key enzymatic activities (including AChE, GABA-T, and digestive enzymes) and the induction of severe oxidative imbalance. These findings highlight the dual neurotoxic and oxidative impacts of these botanical agents. A critical finding of this research is the superior performance of nanoemulsion formulations, which achieved a several-fold increase in larvicidal potency compared to crude extracts. The physicochemical characterization verified that the stability and diminutive droplet size (10–19 nm) of these nano-systems enhance the effective delivery and infiltration of bioactive molecules into larval tissues. Furthermore, the effectiveness of methanol extracts over hexane and aqueous solvents demonstrates the importance of solvent polarity in maximizing the extraction of potent bioactive metabolites. Comprehensive GC-MS and UPLC-MS/MS profiling revealed a marked phytochemical diversity. While both plants share common classes such as flavonoids and fatty acids, *C. cinerariaefolium* was uniquely distinguished by the presence of pyrethrins, aurones, and triterpenoids, which explains its higher toxicological impact. These findings validate the capacity of both plants as enduring sources of bioactive natural products and illustrate that the amalgamation of green nanotechnology with botanical extracts offers an effective, environmentally sustainable approach for mosquito vector management.

## Figures and Tables

**Table 1:** The percent mortality of *Culex pipiens* larvae treated with *Chrysanthemum cinerariaefolium* and *Oenothera biennis* extracts, 24 h post-treatment.

Plant Type	Solvent	Concentration (ppm)						
		0	25	50	125	250	500	1000
*C. cinerariaefolium*	Methanol	0 ± 0 ^aF^	16.00 ± 2.31 ^bE^	29.33 ± 1.33 ^bD^	53.33 ± 2.67 ^bC^	76.00 ± 2.31 ^bB^	100 ± 0.00 ^aA^	100.0 ± 0.00 ^aA^
	Hexane	0 ± 0 ^aG^	12.00 ± 0.00 ^cF^	20.00 ± 2.31 ^cE^	28.00 ± 2.31 ^cD^	46.67 ± 3.53 ^cC^	85.33 ± 2.67 ^bB^	100.0 ± 0.00 ^aA^
	Water	0 ± 0 ^aG^	2.67 ± 1.33 ^dF^	8.00 ± 2.31 ^dE^	13.33 ± 1.33 ^dD^	24.00 ± 2.31 ^dC^	38.67 ± 3.53 ^cB^	74.67 ± 3.53 ^bA^
	Nano	0 ± 0 ^aE^	28.00 ± 2.31 ^aD^	45.33 ± 2.67 ^aC^	90.67 ± 3.53 ^aB^	100.0 ± 0.00 ^aA^	100.0 ± 0.00 ^aA^	100.0 ± 0.00 ^aA^
	Temephos	82.7						
*O. biennis*	Methanol	0 ± 0 ^aG^	10.67 ± 1.33 ^bF^	18.67 ± 1.33 ^bE^	26.67 ± 1.33 ^bD^	45.33 ± 2.67 ^bC^	80.00 ± 0.00 ^bB^	100.0 ± 0.00 ^aA^
	Hexane	0 ± 0 ^aG^	4.00 ± 0.00 ^cF^	13.33 ± 1.33 ^cE^	20.00 ± 2.31 ^cD^	30.67 ± 1.33 ^cC^	61.33 ± 1.33 ^cB^	85.33 ± 2.67 ^bA^
	Water	0 ± 0 ^aG^	1.33 ± 1.33 ^dF^	5.33 ± 1.33 ^dE^	10.67 ± 1.33 ^dD^	21.33 ± 1.33 ^dC^	34.67 ± 1.33 ^dB^	68.0 ± 2.31 ^cA^
	Nano	0 ± 0 ^aE^	18.67 ± 1.33 ^aD^	37.33 ± 1.33 ^aC^	70.67 ± 3.53 ^aB^	100.0 ± 0.00 ^aA^	100.0 ± 0.00 ^aA^	100.0 ± 0.00 ^aA^
	Temephos	100.0						

Lowercase letter: There is no significant difference (*p* > 0.05) between any two means for each plant extract; means within the same column have the same superscript letter. Capital letter: There is no significant difference (*p* > 0.05) between any two means; means within the same row have the same superscript letter. Temephos: 0.25 µg/mL.

**Table 2:** The percent mortality of *Culex pipiens* larvae treated with *Chrysanthemum cinerariaefolium* and *Oenothera biennis* extracts, 48 h post-treatment.

Plant Type	Solvent	Concentration (ppm)						
		0	25	50	125	250	500	1000
*C. cinerariaefolium*	Methanol	0 ± 0 ^aE^	25.33 ± 2.67 ^bD^	50.67 ± 1.33 ^bC^	86.67 ± 6.67 ^bB^	100 ± 0.00 ^abA^	100 ± 0.00 ^aA^	100 ± 0.00 ^aA^
	Hexane	0 ± 0 ^aF^	22.67 ± 1.33 ^cE^	40.00 ± 4.00 ^cD^	56.00 ± 2.31 ^cC^	72.00 ± 2.31 ^cB^	100 ± 0.00 ^aA^	100 ± 0.00 ^aA^
	Water	0 ± 0 ^aG^	8.00 ± 2.31 ^dF^	18.67 ± 1.33 ^dE^	32.00 ± 2.31 ^dD^	41.33 ± 3.53 ^dC^	74.67 ± 2.67 ^bB^	90.67 ± 4.81 ^bA^
	Nano	0 ± 0 ^aD^	45.33 ± 2.67 ^aC^	89.33 ± 1.33 ^aB^	100.0 ± 0.00 ^aA^	100.0 ± 0.00 ^aA^	100.0 ± 0.00 ^aA^	100.0 ± 0.00 ^aA^
	Temephos	82.7						
*O. biennis*	Methanol	0 ± 0 ^aF^	17.33 ± 1.33 ^bE^	37.33 ± 1.33 ^bD^	56.00 ± 2.31 ^bC^	73.33 ± 3.53 ^bB^	100 ± 0.00 ^aA^	100.0 ± 0.00 ^aA^
	Hexane	0 ± 0 ^aG^	13.33 ± 1.33 ^cF^	20.00 ± 2.31 ^cE^	41.33 ± 2.67 ^cD^	56.00 ± 2.31 ^cC^	81.33 ± 1.33 ^bB^	100.0 ± 0.00 ^aA^
	Water	0 ± 0 ^aG^	4.00 ± 0.00 ^dF^	10.67 ± 1.33 ^dE^	18.67 ± 1.33 ^dD^	28.00 ± 2.31 ^dC^	54.67 ± 1.33 ^cB^	80.0 ± 0.00 ^bA^
	Nano	0 ± 0 ^aF^	34.67 ± 1.33 ^aE^	76.00 ± 2.31 ^aD^	98.67 ± 1.33 ^aC^	100.0 ± 0.00 ^aA^	100.0 ± 0.00 ^aA^	100.0 ± 0.00 ^aA^
	Temephos	100.0						

Lowercase letter: There is no significant difference (*p* > 0.05) between any two means for each plant extract; means within the same column have the same superscript letter. Capital letter: There is no significant difference (*p* > 0.05) between any two means; means within the same row have the same superscript letter. Temephos: 0.25 µg/mL.

**Table 3:** Lethal concentrations (ppm) of *Chrysanthemum cinerariaefolium* extracts on *Culex pipiens* mortality, 24 and 48 h post-treatment.

Time (h)	Treatment	LC_50_ (Low–Up)	LC_90_ (Low–Up)	LC_95_ (Low–Up)	Slope ± SE	X^2^ (Sign.)
24	Methanol	94.81 (80.32–111.49)	482.31 (371.78–677.52)	764.88 (559.07–1160.23)	1.814 ± 0.146	6.623 (0.157)
	Hexane	163.31 (86.06–312.36)	803.16 (640.98–2933.41)	1261.56 (1095.73–5720.75)	1.852 ± 0.125	28.112 (0.000)
	Water	557.56 (384.63–1028.22)	3912.12 (2561.88–14,751.22)	6796.19 (4261.94–32,294.73)	1.514 ± 0.131	9.952 (0.041)
	Nano	43.89 (32.43–56.15)	111.40 (90.03–184.05)	145.06 (116.35–257.86)	3.168 ± 0.291	9.700 (0.045)
48	Methanol	44.46 (39.25–49.85)	115.22 (98.07–142.67)	150.92 (124.38–195.50)	3.098 ± 0.282	2.853 (0.582)
	Hexane	73.90 (42.10–113.07)	367.19 (267.08–837.52)	578.44 (432.84–1539.15)	1.840 ± 0.130	17.207 (0.001)
	Water	209.99 (143.36–318.23)	1342.25 (928.32–3114.13)	2270.97 (1519.93–6165.80)	1.590 ± 0.117	10.148 (0.038)
	Nano	26.63 (23.40–29.43)	49.90 (44.22–59.51)	59.62 (51.47–74.78)	4.699 ± 0.602	0.249 (0.992)

**Table 4:** Lethal concentrations (ppm) of *Oenothera biennis* extracts on *Culex pipiens* mortality, 24 and 48 h post-treatment.

Time (h)	Treatment	LC_50_ (Low–Up)	LC_90_ (Low–Up)	LC_95_ (Low–Up)	Slope ± SE	X^2^ (Sign.)
24	Methanol	178.43 (98.62–331.77)	894.26 (701.00–3021.51)	1412.20 (1182.75–5840.66)	1.830 ± 0.125	25.200 (0.000)
	Hexane	324.67 (223.95–518.57)	1989.70 (1372.09–5170.61)	3326.43 (2222.33–10,245.06)	1.627 ± 0.124	10.575 (0.031)
	Water	673.26 (544.81–875.79)	4393.02 (2837.50–8092.98)	7476.09 (4481.50–15,367.71)	1.573 ± 0.142	5.926 (0.204)
	Nano	58.28 (51.70–65.41)	158.85 (134.64–196.37)	211.07 (173.50–272.96)	2.943 ± 0.236	7.840 (0.097)
48	Methanol	80.15 (54.53–110.38)	361.93 (267.60–617.49)	554.92 (405.92–1041.06)	1.957 ± 0.127	10.972 (0.026)
	Hexane	140.69 (88.62–219.87)	753.69 (547.02–1754.97)	1212.89 (884.27–3277.14)	1.758 ± 0.122	14.883 (0.004)
	Water	393.22 (327.44–484.19)	2652.59 (1839.53–4322.74)	4557.00 (2955.47–8162.70)	1.545 ± 0124	7.588 (0.107)
	Nano	31.89 (28.23–35.36)	66.74 (58.37–80.39)	82.27 (69.97–103.97)	3.997 ± 0.436	1.215 (0.875)

**Table 5:** The effect of *Chrysanthemum cinerariaefolium* and *Oenothera biennis* extracts and nanoemulsions on the activities of different esterase enzymes in mosquitoes.

**Plant Extract**	Acetylcholinesterase (AChE) (nmol ACh Hydrolyzed/min/mg Protein)	α-Esterase (α-EST) (mmol/min/mg Protein)	*β*-Esterase (*β*-EST) (mmol/min/mg Protein)
Control	9.44 ± 0.04 ^a^	2.27 ± 0.03 ^a^	3.05 ± 0.07 ^a^
*C. cinerariaefolium* (extract)	5.25 ± 0.02 ^d^	1.26 ± 0.02 ^bc^	1.70 ± 0.04 ^bc^
*O. biennis* (extract)	7.86 ± 0.28 ^b^	2.04 ± 0.09 ^a^	2.78 ± 0.15 ^a^
*C. cinerariaefolium* (nanoemulsion)	2.59 ± 0.22 ^e^	0.95 ± 0.07 ^c^	1.27 ± 0.09 ^c^
*O. biennis* (nanoemulsion)	6.21 ± 0.10 ^c^	1.50 ± 0.20 ^b^	1.98 ± 0.26 ^b^

Lowercase letter: There is no significant difference (*p* > 0.05) between any two means; means within the same column have the same superscript letter.

**Table 6:** The effect of *Chrysanthemum cinerariaefolium* and *Oenothera biennis* extracts and nanoemulsions on the activities of infection response enzymes in mosquitoes.

**Plant Extract**	Gamma-Aminobutyric Acid Transaminase (GABA-T) (mg/L)	Amylase (Units/g Tissue)	Lipase (Units/g Tissue)
Control	4.50 ± 0.02 ^a^	19.30 ± 0.10 ^a^	1.90 ± 0.02 ^a^
*C. cinerariaefolium* (extract)	2.52 ± 0.01 ^c^	10.73 ± 0.05 ^d^	1.05 ± 0.01 ^d^
*O. biennis* (extract)	3.80 ± 0.19 ^b^	17.74 ± 0.35 ^b^	1.66 ± 0.10 ^b^
*C. cinerariaefolium* (nanoemulsion)	1.79 ± 0.12 ^d^	7.67 ± 0.04 ^e^	0.80 ± 0.02 ^e^
*O. biennis* (nanoemulsion)	2.76 ± 0.20 ^c^	14.94 ± 0.28 ^c^	1.40 ± 0.04 ^c^

Lowercase letter: There is no significant difference (*p* > 0.05) between any two means; means within the same column have the same superscript letter.

**Table 7:** The effect of *Chrysanthemum cinerariaefolium* and *Oenothera biennis* extracts on markers of the antioxidant defense in mosquitoes.

Plant Extract	SOD (Units/g Tissue)	G-S-T (Units/g Tissue)	CAT (U/mg Protein/min)	GSH (µg/mg Protein)	LPO (nmol/mg Protein)	TPC (nmol/mg Protein)
Control	2.42 ± 0.03 ^a^	56.41 ± 0.59 ^a^	3.65 ± 0.04 ^a^	1.66 ± 0.03 ^a^	3.50 ± 0.03 ^c^	5.42 ± 0.03 ^d^
*C. cinerariaefolium* (extract)	31.37 ± 0.33 ^d^	2.03 ± 0.02 ^c^	0.92 ± 0.02 ^c^	6.17 ± 0.05 ^b^	9.56 ± 0.05 ^b^	9.56 ± 0.05 ^a^
*O. biennis* (extract)	49.59 ± 2.23 ^b^	3.24 ± 0.30 ^a^	1.61 ± 0.02 ^a^	3.50 ± 0.03 ^c^	5.62 ± 0.16 ^d^	5.43 ± 0.03
*C. cinerariaefolium* (nanoemulsion)	24.69 ± 0.74 ^e^	1.64 ± 0.02 ^c^	0.72 ± 0.01 ^d^	12.16 ± 0.10 ^a^	15.09 ± 0.09 ^a^	15.09 ± 0.09 ^ab^
*O. biennis* (nanoemulsion)	37.98 ± 2.35 ^c^	2.72 ± 0.14 ^b^	1.31 ± 0.03 ^b^	5.86 ± 0.32 ^b^	8.90 ± 0.35 ^c^	8.57 ± 0.05 ^ac^

Lowercase letter: There is no significant difference (*p* > 0.05) between any two means; means within the same column have the same superscript letter.

**Table 8:** GC/MS identified metabolites from *Chrysanthemum cinerariaefolium* flowers *n*-hexane extract.

No.	Component	R_t_ (min.)	RI		% Composition	Mol. Formula	Identification Method
			Cal.	Rep.			
1	Hemimellitene	8.095	970	979	0.80	C_9_H_12_	RI, MS
2	α-Cubebene	19.974	1334	1332	0.27	C_15_H_24_	RI, MS
3	*β*-Cubebene	21.013	1371	1372	0.72	C_15_H_24_	RI, MS
4	3(10)-Caren-4-ol, acetoacetic acid ester	21.576	1392	-	0.11	C_14_H_20_O_3_	RI, MS
5	*epi*-*β*-Caryophyllene	22.870	1441	1455	1.19	C_15_H_24_	RI, MS
6	α-Guaiene	23.616	1469	1469	2.05	C_15_H_24_	RI, MS
7	(+)-Ledene	23.813	1477	1478	2.90	C_15_H_24_	RI, MS
8	α-Amorphene	24.395	1499	1494	0.36	C_15_H_24_	RI, MS
9	*O*-Menth-8-ene-4-methanol	24.897	1519	1519	0.51	C_15_H_26_O	RI, MS
10	Viridiflorol	26.015	1564	1568	1.07	C_15_H_26_O	RI, MS
11	*β*-Oplopenone	26.208	1572	1573	0.32	C_15_H_24_O	RI, MS
12	α,α-dimethyl-1-vinyl-*O*-Menth-8-ene-4-methanol	26.835	1597	-	0.31	C_15_H_26_O	RI, MS
13	(1-pentylhexyl)-Benzene	27.088	1608	-	0.75	C_17_H_28_	RI, MS
14	Eudesm-4(14)-en-11-ol	27.362	1619	1618	3.34	C_15_H_26_O	RI, MS
15	ent-Germacra-4(15),5,10(14)-trien-1*β*-ol	28.072	1650	-	0.31	C_15_H_24_O	RI, MS
16	α-Oplopanone	29.049	1691	1695	2.15	C_15_H_26_O_2_	RI, MS
17	(3,3-dimethyldecyl)-Benzene	29.339	1704	-	1.07	-	RI, MS
18	*trans*-*Z*-α-Bisabolene epoxide	30.555	1759	-	0.13	C_15_H_24_O	RI, MS
19	Isospathulenol	30.695	1765	-	0.10	C_15_H_24_O	RI, MS
20	Neophytadiene	31.970	1823	-	1.98	C_20_H_38_	RI, MS
21	[R-[R*,R*-(E)]]-3,7,11,15-tetramethyl-2-Hexadecene	32.175	1833	1830	0.24	C_20_H_40_	RI, MS
22	(1-ethylundecyl)-Benzene	32.399	1843	-	0.36	C_19_H_32_	RI, MS
23	Nonadecane	33.303	1886	1900	0.66	C_19_H_40_	RI, MS
24	Hexadecanoic acid, methyl ester	33.486	1894	-	0.62	C_17_H_34_O_2_	RI, MS
25	2-Methyltetracosane	33.710	1905	-	0.30	C_25_H_52_	RI, MS
26	Eicosane	33.968	1918	-	1.33	C_20_H_42_	RI, MS
27	*n*-Hexadecanoic acid	34.485	1944	1950	0.82	C_16_H_32_O_2_	RI, MS
28	1-Nonadecene	35.136	1976	-	0.23	C_19_H_38_	RI, MS
29	Heneicosane	35.315	1985	-	1.75	C_21_H_44_	RI, MS
30	(*Z*,*Z*,*Z*)-9,12,15-Octadecatrienoic acid	36.294	2034	-	0.13	C_18_H_30_O_2_	RI, MS
31	(*Z*,*Z*)-9,12-Octadecadienoic acid, methyl ester	36.698	2055	-	0.66	C_19_H_34_O_2_	RI, MS
32	11-Octadecenoic acid, methyl ester	36.875	2064	-	0.13	C_19_H_36_O_2_	RI, MS
33	2-Nonadecanone	36.990	2070	-	0.20	C_19_H_38_O	RI, MS
34	Methyl *γ*-linolenate	39.455	2202	-	0.36	C_19_H_32_O_2_	RI, MS
35	*cis*-5,8,11,14,17-Eicosapentaenoic acid	40.175	2242	-	0.56	-	RI, MS
36	Caryophyllenyl alcohol	40.619	2267	-	0.62	-	RI, MS
37	Tetracosane	40.912	2284	-	4.06	C_24_H_50_	RI, MS
38	4,8,12,16-Tetramethylheptadecan-4-olide	41.242	2302	-	0.24	C_21_H_40_O_2_	RI, MS
39	Hexanedioic acid, *bis*(2-ethylhexyl) ester	42.187	2358	-	5.25	C_22_H_42_O_4_	RI, MS
40	*n*-Tetracosanol-1	42.455	2374	-	0.28	C_24_H_50_O	RI, MS
41	1-Heptatriacotanol	43.757	2452	-	0.22	C_37_H_76_O	RI, MS
42	2,6,10,14-tetramethyl-Pentadecane	45.447	2557	-	0.53	C_19_H_40_	RI, MS
43	Tetracontane	47.408	2685	-	5.74	C_40_H_82_	RI, MS
44	1,3-Benzenedicarboxylic acid, *bis*(2-ethylhexyl) ester	47.651	2701	-	0.78	C_24_H_38_O_4_	RI, MS
45	Tetratetracontane	48.813	2781	-	3.04	C_44_H_90_	RI, MS
46	Hexatriacontane	50.315	2886	-	10.81	C_36_H_74_	RI, MS
47	17-Pentatriacontene	52.646	3056	-	0.89	C_35_H_70_	RI, MS
48	*β*-Amyrin	56.269	3321	-	1.73	C_30_H_50_O	RI, MS
49	24-Norursa-3,12-diene	56.980	3373	-	1.54	C_29_H_46_	RI, MS
**% Identification**					**64.52**		

**Table 9:** GC/MS identified metabolites from *Oenothera biennis* flowers *n*-hexane extract.

No.	Component	R_t_ (min.)	RI		% Composition	Mol. Formula	Identification Method
			Cal.	Rep.			
1	1-ethyl-3-methyl-Benzene	7.840	939	-	0.30	C_9_H_12_	RI, MS
2	1,2,3-trimethyl-Benzene	8.095	947	-	1.05	C_9_H_12_	RI, MS
3	Caryophyllene	23.477	1464	-	0.85	C_15_H_24_	RI, MS
4	Aromadendrene	23.754	1474	-	0.93	C_15_H_24_	RI, MS
5	1-Pentadecene	23.856	1478	-	0.84	C_15_H_30_	RI, MS
6	Heneicosane	24.154	1490	-	7.11	C_21_H_44_	RI, MS
7	Globulol	24.875	1518	-	0.27	C_15_H_26_O	RI, MS
8	Eicosane	25.262	1534	-	4.42	C_20_H_42_	RI, MS
9	Viridiflorol	25.976	1563	-	0.48	C_15_H_26_O	RI, MS
10	Tetradecanal	26.434	1581	-	3.94	C_14_H_28_O	RI, MS
11	Hexadecane	26.595	1588	1600	0.23	C_16_H_34_	RI, MS
12	(*E*)-10-Heptadecen-8-ynoic acid, methyl ester	26.815	1596	-	0.27	C_18_H_30_O_2_	RI, MS
13	Sclareol	27.290	1616	-	0.85	C_15_H_26_O	RI, MS
14	Cubenol	27.395	1621	1642	0.10	C_15_H_26_O	RI, MS
15	2-Pentadecanone	28.461	1666	-	0.39	C_15_H_30_O	RI, MS
16	Pristane	28.726	1678	-	0.32	C_19_H_40_	RI, MS
17	*α*-Eudesmane	28.995	1689		1.15	C_20_H_36_O	RI, MS
18	1-Nonadecene	30.955	1776	-	0.16	C_19_H_38_	RI, MS
19	Hexadecanal	31.087	1782	-	0.44	C_16_H_32_O	RI, MS
20	Hexahydrofarnesyl Acetone	31.788	1815	-	0.36	C_18_H_36_O	RI, MS
21	Neophytadiene	31.950	1822	1837	1.74	C_20_H_38_	RI, MS
22	[R-[R*,R*-(*E*)]]-2-Hexadecene, 3,7,11,15-tetramethyl-,	32.155	1832	1844	0.20	C_20_H_40_	RI, MS
23	Octacosane	33.295	1886	-	1.04	C_28_H_58_	RI, MS
24	Methyl Palmitate	33.479	1894	-	1.82	C_17_H_34_O_2_	RI, MS
25	Palmitic Acid	34.278	1933	1959	0.28	C_16_H_32_O_2_	RI, MS
26	Ethyl Palmitate	34.869	1963	-	0.64	C_18_H_36_O_2_	RI, MS
27	14-methyl-Hexadecanoic acid methyl ester	35.486	1993	-	0.15	C_18_H_36_O_2_	RI, MS
28	3-hydroxy-Hexadecanoic acid methyl ester	36.294	2034	-	0.29	C_17_H_34_O_3_	RI, MS
29	Methyl Linoleate	36.688	2055	-	1.27	C_19_H_34_O_2_	RI, MS
30	Methyl *cis*-vaccenate	36.875	2064	-	0.69	C_19_H_36_O_2_	RI, MS
31	2-Nonadecanone	36.975	2070	-	0.13	C_19_H_38_O	RI, MS
32	Methyl stearate	37.433	2093	-	0.66	C_19_H_38_O_2_	RI, MS
33	Ethyl Stearate	38.695	2161	-	0.39	C_20_H_40_O_2_	RI, MS
34	Octadecanal	39.195	2188	-	0.15	C_18_H_36_O	RI, MS
35	Methyl *γ*-linolenate	40.075	2237	-	0.40	C_19_H_32_O_2_	RI, MS
36	3-(4-hydroxybutyl)-2-methyl-Cyclohexanone	40.601	2266	-	0.25	C_11_H_20_O_2_	RI, MS
37	*iso*-Arachidic acid methyl ester	41.055	2292	-	0.14	C_21_H_42_O_2_	RI, MS
38	4,8,12,16-Tetramethylheptadecan-4-olide	41.215	2301	-	0.34	C_21_H_40_O_2_	RI, MS
39	Tetracontane	41.795	2335	-	20.74	C_40_H_82_	RI, MS
40	Tetracosane	47.349	2681	-	3.98	C_24_H_50_	RI, MS
41	Tetratetracontane	48.510	2760	-	1.05	C_34_H_70_	RI, MS
42	Tetratriacontane	48.785	2779	-	0.80	C_34_H_70_	RI, MS
43	Humulane-1,6-dien-3-ol	50.465	2897	-	0.35	C_15_H_26_O	RI, MS
44	1,4-dimethyl-8-*iso*-propylidenetricyclo[5.3.0.0(4,10)]decane	50.955	2933	-	0.63	C_15_H_24_	RI, MS
45	3-methyl-Nonacosane	51.221	2952		0.15	C_30_H_62_	RI, MS
46	Hexatriacontane	52.987	3081		13.79	C_36_H_74_	RI, MS
47	(3*β*)-Lanost-8-en-3-ol (Lanosterol)	54.756	3211	3200	0.29	C_30_H_50_O	RI, MS
48	(3*β*)-Olean-12-en-3-ol, acetate (*β*-Amyrin acetate)	56.188	3316	-	0.47	C_32_H_52_O_2_	RI, MS
49	3,8,8,11a-Tetramethyldodecahydro-5H-3,5a-epoxynaphtho[2, 1-c]oxepine	56.896	3367	-	0.47	C_18_H_30_O_2_	RI, MS
50	1-Heptatriacotanol	58.055	3452	-	0.27	C_37_H_76_O	RI, MS
% Identification					78.03		

## Data Availability

The datasets used and/or analyzed during the current study are available from the corresponding author upon reasonable request. Correspondence: mohamed.albaz@fsc.bu.edu.eg.

## Supplementary Materials

The following supporting information can be downloaded at: https://www.mdpi.com/article/10.3390/insects17090925/s1, Table S1: UPLC/MS identified metabolites from *Chrysanthemum cinerariaefolium* methanol, ethyl acetate and aqueous extracts; Table S2: UPLC/MS identified metabolites from *Oenothera biennis* methanol, ethyl acetate and aqueous extracts.
