# Supplementary material for: Effect of Nanoemulsions of Chrysanthemum cinerariaefolium and Oenothera biennis Flowers Against the West Nile Virus Vector, Culex pipiens, with Phytochemical Profile
Source: Insects. 2026 Sep 3;17(9):925. doi: 10.3390/insects17090925 (PMC13607256; doi:10.3390/insects17090925)
Supplement: Supplementary file 1 [file insects-17-00925-s001.zip › insects-4518965-supplementary.pdf]

Table S1. UPLC/MS identified metabolites from *Chrysanthemum cinerariaefolium* methanol, ethyl acetate and aqueous extracts

No.	Components	Class	Mol. Formula	R_t (min.)	$[M-H]^-$ *m/z*	$[M+H]^+$ *m/z*	% Composition			Ref.
							CW-Me	CW-Eth. Ac.	CW-Aq.	
1	*tetra*-Hydroxy-*tri*-methoxy-*di*-hydroxyflavone	Flavonoid	$C_{19}H_{18}O_{10}$	0.84	**377**	**381**	**8.25**<br>**(32.51)**	**5.19**<br>**(21.37)**	-	[42]
2	Chlorogenic acid[c]	Cinnamic acid deriv.	$C_{16}H_{18}O_9$	2.74	**353**	**-**	**3.59**	0.66	-	[43-45]
3	Kaempferol-galloyl-hexoside	Flavonoid	$C_{28}H_{24}O_{15}$	3.32	**599**	**-**	-	-	**6.08**	[46]
4	Caffeic acid	Phenolic acid	$C_9H_8O_4$	4.01	**179**	**-**	-	-	**2.53**	[44]
5	Apigenin-hexoside[c]	Flavonoid	$C_{21}H_{20}O_{10}$	4.04	**431**	**-**	0.48	0.54	-	[43-45]
6	*trans*-Vaccenic acid[c]	Fatty acid	$C_{18}H_{34}O_2$	4.67	**281**	**-**	0.38	-	1.68	[45]
7	Cirsimaritin[c]	Flavonoid	$C_{17}H_{14}O_6$	5.61	**313**	**-**	1.83	-	**2.21**	[45]
8	Quercetin-rutinoside (rutin)[c]	Flavonoid	$C_{27}H_{30}O_{16}$	6.00	**609**	**771**	**3.73**<br>(0.79)	1.07	1.90	[44,47]
9	Luteolin-hexuronyl-hexoside[c]	Flavonoid	$C_{27}H_{28}O_{17}$	6.35<br>**6.60**	**623**	**625**	**6.95**<br>**(6.50)**	**3.44**<br>**(7.63)**	-	[48]
10	*di*-Caffeoyl-quinic acid[c]	Cinnamic acid deriv.	$C_{25}H_{24}O_{12}$	6.89<br>7.06	**515**	**-**	**7.00**	1.73	-	[49]
11	Isorhamnetin-hexoside[c]	Flavonoid	$C_{22}H_{22}O_{12}$	6.91	**477**	**-**	-	0.92	-	[44]
12	Luteolin-hexoside[c]	Flavonoid	$C_{21}H_{20}O_{11}$	7.10	**447**	**-**	-	-	**2.33**	[43,44]
13	Chrysoeriol[c]	Flavonoid	$C_{16}H_{12}O_6$	7.53	**299**	**-**	**8.52**	**9.68**	-	[47]
14	Acacetin-hexoside[c]	Flavonoid	$C_{21}H_{20}O_9$	8.28	**445**	**-**	0.74	-	-	[50,51]
15	Daidzein-hexoside (puerarin)	Flavonoid	$C_{21}H_{20}O_9$	8.52	**415**	**-**	0.91	1.13	-	[52]
16	*di*-Hydroxy-*oxo*-octadecenoic acid[c]	Oxylipin	$C_{18}H_{32}O_5$	8.97	**327**	**-**	**3.89**	**5.51**	-	[45]
17	*mono*-Hydroxyl-*mono*-methoxy-ursolic acid	Triterpenoid	$C_{31}H_{50}O_5$	9.52	**501**	**503**	**4.37**<br>(1.61)	-	-	[42,53]
18	Pyrethrin I[c]	Pyrethrins	$C_{21}H_{28}O_3$	9.54	**329**	**-**	-	**4.92**	**2.06**	[54]
19	*tetra*-Hydroxy-Flavanone	Flavonoid	$C_{15}H_{12}O_6$	9.89	**287**	**-**	0.33	-	-	[55]
20	Acacetin-malonyl-hexoside[c]	Flavonoid	$C_{25}H_{24}O_{13}$	10.66	**531**	**-**	0.63	-	-	[42]
21	Linolenic acid-hydroperoxide	Fatty acid	$C_{18}H_{30}O_4$	11.91<br>12.27	**309**	**-**	1.08<br>0.73	0.75	-	[56]
22	*tetra*-Hydroxy-*di*-methoxyflavone[c]	Flavonoid	$C_{17}H_{14}O_8$	12.85	**345**	**-**	1.39	1.38	-	[45]

No.	Components	Class	Mol. Formula	R_t (min.)	$[M-H]^-$ *m/z*	$[M+H]^+$ *m/z*	% Composition			Ref.
							CW-Me	CW-Eth. Ac.	CW-Aq.	
23	Phenethyl sophoroside[c]	Phenyl ethanoid glycoside	$C_{20}H_{30}O_{11}$	12.98	**-**	**439**	(3.81)	-	-	[45]
24	Cafestol[c]	Diterpenoid	$C_{20}H_{28}O_3$	13.24	**-**	**317**	(0.50)	-	-	[45]
25	Apigenin[c]	Flavonoid	$C_{15}H_{10}O_5$	14.28	**269**	**277**	0.37 (0.46)	-	-	[57]
26	*tri*-Methoxy-cinnamoyl-quinic acid	Cinnamic acid deriv.	-----	14.31	**411**	**-**	-	0.74	-	[58]
27	*oxo*-Octadecadienoic acid	Fatty acid	$C_{18}H_{30}O_3$	14.55	**293**	**-**	**12.55**	**21.26**	-	[59]
28	Hexopyranosyloxy-hydroxy-propyl ester, octadecatrienoic acid[c]	Fatty acid	$C_{27}H_{46}O_9$	15.33	**559**	**-**	0.89	1.14	-	[45]
29	Hydroxy-octadecadienoic acid[c]	Fatty acid	$C_{18}H_{32}O_3$	15.74	**295**	**279**	**7.33 (3.03)**	**12.66**	-	[45]
30	Proanthocyanidin dimer	Tannin	$C_{30}H_{26}O_{12}$	16.59	**561**	**-**	0.96	-	-	[60]
31	α-Hydroxy-deoxy-cholic acid	Steroid	$C_{24}H_{40}O_4$	17.55	**391**	**-**	0.19	-	-	[61]
32	Naringenin[c]	Flavonoid	$C_{15}H_{12}O_5$	19.16	**271**	**-**	**4.05**	**3.28**	-	[45,47]
33	Linolenic acid	Fatty acid	$C_{18}H_{30}O_2$	19.85	**277**	**-**	1.48	**2.57**	-	[62]
34	Caffeoyl quinate shikmate derivative	Cinnamic acid deriv.	-----	21.06	**509**	**-**	-	1.06	-	[63]
35	Octadecadienoic acid	Fatty acid	$C_{18}H_{32}O_2$	21.69	**279**	**-**	1.59	**2.76**	-	[62]
36	Pinocembrin[c]	Flavonoid	$C_{15}H_{12}O_4$	23.54	**255**	**-**	0.62	1.16	-	[44]
37	Bracteatin[c]	Aurone	$C_{15}H_{10}O_7$	24.26	**-**	**303**	(0.77)	(1.08)	-	[45]
38	Hydroxy-methoxy-methylenedioxy-dimethyl homoisoflavanone	Flavonoid	$C_{21}H_{22}O_6$	29.39	**355**	**-**	0.49	-	-	[64]
	**No. of identified components**						**32**	**23**	**7**	
	**% Identification**									
	ESI negative mode						**85.32**	**83.55**	**18.79**	
	ESI positive mode						**49.98**	**30.08**	**-**	

[C] for the metabolites previously reported from genus *Chrysanthemum*.

Table S2. UPLC/MS identified metabolites from *Oenothera biennis* methanol, ethyl acetate and aqueous extracts

No.	Components	Class	Mol. Formula	R_t (min.)	$[M-H]^-$ *m/z*	$[M+H]^+$ *m/z*	% Composition			Ref.
							OBF-Me	OBF-Eth. Ac.	OBF-Aq.	
1	*tetra*-Hydroxy-*tri*-methoxy-*di*-hydroxyflavone	Flavonoid	$C_{19}H_{18}O_{10}$	0.84	**377**	**381**	**6.46 (33.07)**	**4.39 (13.77)**	-	[42]
2	Apigenin[o]	Flavonoid	$C_{15}H_{10}O_5$	0.88	**269**	**-**	-	-	**8.91**	[65]
3	Catechin-caffeic adduct	Tannin	-----	1.50	**467**	**-**	**3.63**	**4.79**	**7.39**	[66]
4	Catechin hexoside	Tannin	$C_{21}H_{24}O_{11}$	2.32	**451**	**-**	-	-	**2.33**	[67]
5	Caffeoyl hexoside	Cinnamic acid deriv.	$C_{15}H_{18}O_9$	2.76	**341**	**-**	-	-	1.21	[68]
6	Chlorogenic acid[o]	Cinnamic acid deriv.	$C_{16}H_{18}O_9$	2.86	**353**	**355**	**2.18 (2.36)**	-	0.60	[69]
7	*di*-hydro-Caffeic acid	Cinnamic acid deriv.	$C_9H_{10}O_4$	3.40	**181**	**-**	-	-	**5.43**	[70]
8	*di*-homo-γ-Linolenic acid[o]	Fatty acid	$C_{20}H_{34}O_2$	3.84	**-**	**307**	(0.94)	-	-	[71]
9	Quercetin-pentoside (quercitrin)[o]	Flavonoid	$C_{21}H_{20}O_{11}$	4.08	**447**	**-**	-	-	**4.20**	[72]
10	Apigetrin[o]	Flavonoid	$C_{21}H_{20}O_{10}$	4.46	**431**	**-**	1.53	-	1.21	[73]
11	(*epi*) Gallocatechin	Tannin	$C_{15}H_{14}O_7$	4.55	**305**	**-**	-	-	**3.18**	[68,74]
12	Quercitrin-*di*-hexoside	Flavonoid	$C_{27}H_{30}O_{17}$	5.45	**771**	**-**	-	-	0.46	[75,76]
13	Quercetin hexuronyl-hexoside[o]	Flavonoid	$C_{27}H_{28}O_{18}$	5.68	**639**	**-**	-	-	1.21	[71]
14	Rutin[o]	Flavonoid	$C_{27}H_{30}O_{16}$	6.11	**609**	**-**	1.34	0.95	1.81	[65]
15	Isorhamnetin-rutinoside	Flavonoid	$C_{28}H_{32}O_{16}$	6.41	**623**	**625**	**6.55** (1.45)	**3.15 (3.09)**	**3.24**	[77,78]
16	*di*-Caffeoyl-quinic acid	Cinnamic acid deriv.	$C_{25}H_{24}O_{12}$	7.02	**515**	**565**	(1.17)	1.30 (0.28)	-	[49]
17	Kaempferol-hexuronide[o]	Flavonoid	$C_{21}H_{18}O_{12}$	7.12	**461**	**-**	1.19	-	-	[71]
18	Rhamnetin-hexuronide	Flavonoid	$C_{22}H_{20}O_{13}$	7.53	**491**	**-**	**4.43**	-	-	[79]
19	Luteolin[o]	Flavonoid	$C_{15}H_{10}O_6$	8.25	**285**	**287**	1.09 (1.13)	-	0.78	[65]
20	*oxo*-*di*-hydroxy-Octadecenoic acid	Fatty acid	$C_{18}H_{32}O_5$	8.99	**327**	**-**	-	1.57	**2.59**	[78,80]
21	Naringenin[o]	Flavonoid	$C_{15}H_{12}O_5$	9.04 19.14	**271**	**273**	**39.83 (8.01)**	**38.52 (4.06)**	1.06	[65]
22	Quercetin-dimethyl ether[o]	Flavonoid	$C_{17}H_{14}O_7$	9.52	**329**	**331**	-	**2.36** (0.96)	**2.12**	[81]

No.	Components	Class	Mol. Formula	R_t (min.)	$[M-H]^-$ *m/z*	$[M+H]^+$ *m/z*	% Composition			Ref.
							OBF-Me	OBF-Eth. Ac.	OBF-Aq.	
23	*tetra*-hydroxy-Flavanone	Flavonoid	$C_{15}H_{12}O_6$	9.88	**287**	**-**	-	-	0.75	[55]
24	Syringetin-hexoside	Flavonoid	$C_{23}H_{24}O_{13}$	10.12	**507**	**-**	-	-	0.92	[52]
25	Caffeic acid pentoside[o]	Cinnamic acid deriv.	$C_{14}H_{16}O_8$	11.47	**311**	**-**	-	-	0.62	[71]
26	*oxo*-Octadecadienoic acid	Fatty acid	$C_{18}H_{30}O_3$	14.52 14.69	**293**	**317**	**6.49 (5.25)**	**14.97 (9.44)**	1.10	[59]
27	Hydroxy-octadecadienoic acid[o]	Fatty acid	$C_{18}H_{32}O_3$	15.72	**295**	**279**	**5.08 (3.46)**	**7.38 (3.65)**	-	[45]
28	α-Hydroxy-deoxy-cholic acid	Steroid	$C_{24}H_{40}O_4$	17.52	**391**	**393**	**9.50 (11.72)**	**11.08 (10.44)**	-	[61]
29	*tetra*-methyl-Ellagic acid[o]	Tannin	$C_{18}H_{14}O_8$	20.24	**357**	**-**	-	1.29	-	[71]
30	Linoleic acid[o]	Fatty acid	$C_{18}H_{32}O_2$	21.62	**279**	**-**	-	1.25	-	[71]
31	Chrysoeriol[o]	Flavonoid	$C_{16}H_{12}O_6$	22.22	**299**	**-**	**3.53**	**4.48**	-	[47]

	OBF-Me	OBF-Eth. Ac.	OBF-Aq.	
**No. of identified components**	**16**	**14**	**21**	
**% Identification**				
ESI negative mode	**92.83**	**97.48**	**51.12**	
ESI positive mode	**68.56**	**45.69**	**-**	

[o]for compounds reported from genus *Oenothera.*
